# A scoping review exploring cancer survivorship in Africa: 2011 to 2024

**DOI:** 10.1007/s11764-025-01805-5

**Published:** 2025-04-25

**Authors:** Tonia Onyeka, Abdulhafiz Zakieh, Isaiah Gitonga, Goodluck Nchasi, Md Ahasanur Rahman, Nikki Prattipati, Alicia A. Livinski, Timiya S. Nolan, Mishka Kohli Cira

**Affiliations:** 1 Department of Anaesthesia/Pain & Palliative Care Unit, College of Medicine, University of Nigeria/IVAN Research Institute, Ituku-Ozalla Campus, Enugu, Nigeria; 2 Pediatric Oncology Branch, National Cancer Institute, National Institutes of Health, Bethesda, MD USA; 3 Maynooth University, Maynooth, Co. Kildare Ireland; 4 Bugando Medical Center, Mwanza, Tanzania; 5 National Cancer Institute, Frederick, MD USA; 6 Birmingham, USA; 7 Present Address: Boston University School of Medicine, Boston, MA USA; 8 National Institutes of Health Library, Office of Research Services, Office of the Director, National Institutes of Health, Bethesda, MD USA; 9 University of Alabama at Birmingham, Birmingham, AL USA; 10 Center for Global Health, National Cancer Institute, Rockville, MD USA

**Keywords:** Cancer, Survivorship, Survivor, Scoping review, Africa

## Abstract

**Purpose:**

The scope of cancer survivorship in Africa remains largely unexplored. This scoping review aims to describe current survivorship-related factors and care delivery characteristics in Africa, and to provide insights that can inform a more systematic integration of survivorship care into the broader cancer care continuum.

**Methods:**

Four databases (PubMed, Embase, Web of Science: Core, CINAHL Plus) were searched for journal articles and grey literature published from 2011 to August 2024 in English. Articles were included if they focused on post-treatment care for African cancer survivors. Covidence® was utilised for screening and data collection. Microsoft Excel was used for data cleaning and analysis.

**Results:**

A total of 9417 articles were screened and 181 included. The number of studies per year grew from 6 (2011) to 32 (2023). These comprised 71 conference abstracts and 110 journal articles conducted in 19 African countries. The study methodologies utilised include 97 quantitative, 42 qualitative, 25 programme descriptions, and 18 mixed methods. Childhood survivorship was the focus for 40 studies. Psychosocial effects of cancer and its treatment were discussed in 104 studies.

**Conclusions:**

Interest in post-treatment cancer survivorship and related care is increasing across Africa. While significant research has explored the physical and psychosocial effects of cancer, there remains a critical need for studies focused on care coordination, targeted interventions, and strategies to improve quality-of-life outcomes for survivors.

**Implications for Cancer Survivors:**

The overall scope of post-treatment cancer survivorship care in Africa primarily centres on psychosocial outcomes, with limited emphasis on care coordination. This underscores the need for further research and the development of context-relevant, feasible, and integrated care models within African health systems.

**Supplementary Information:**

The online version contains supplementary material available at https://doi.org/10.1007/s11764-025-01805-5.

## Introduction

The cancer incidence in Africa has doubled in the past 30 years and is projected to double again by 2040 [1, 2]. This alarming trend is attributed to factors such as rising carcinogenic exposures and lifestyle changes in low- and middle-income countries (LMICs) associated with the epidemiologic shift [3]. Despite the worsening cancer burden in Africa, the landscape of cancer diagnosis, treatment, and survival is improving with advances in healthcare and infrastructure [4]. Hence, a growing number of people are living with, through, and beyond their cancer diagnosis in Africa.

As cancer survival rates improve, cancer survivorship care must address challenges that extend beyond the realm of palliative care. Cancer survivors often grapple with lasting side-effects including physical side effects of cancer treatment, with pain and fatigue being the most common, but also encompassing a wide range of complications including heart and lung issues, and infertility [5]; emotional and psychological distress, including depression and anxiety reported from literature in high-income settings [6, 7], with added impacts of shame and stigma associated with a cancer diagnosis as reported by some qualitative work in Africa [8, 9]; and, financial hardship due to cancer diagnosis and treatment, including medical debt and reduction in income due to the inability to work [10]. To ensure survivors’ well-being and quality of life, there is a need to focus on developing comprehensive and context relevant cancer survivorship programmes in the region that are developed with and responsive to input from people living with and affected by cancer. This requires a multi-sectoral approach that considers the individual, community, facility, and policy levels.

While survivorship is defined by the US National Cancer Institute (NCI) as beginning at the time of cancer diagnosis [11], there are unique considerations in the post-primary treatment phase. These include access to treatment summaries and care plans, financial toxicity, re-entering the workforce, and late- or long-term side effects from cancer or its treatment including physical, psychological, emotional, spiritual, and social aspects. Others are stigma, family or spousal relationships, cancer recurrence, lifestyle and behaviour change for cancer prevention, primary care utilisation and care coordination, rehabilitation, and quality of life [12]. Previous studies have established context-specific definitions of survivorship. Mullen et al. [13] in their global review of National Cancer Control Plans (NCCP) adopted a broad definition, through consensus, of a cancer survivor as ‘an individual from the time of diagnosis through to the end of life’. Their analysis primarily focused on survivorship care in the post-treatment period of care with curative intent (including continuing hormonal therapy) as well as care for those living with metastatic or advanced cancers. Garton et al. [1] examined the inclusion of cancer survivorship-related strategies and objectives in NCCPs across African countries. The review noted the lack of a consistent definition of cancer survivorship and, for their study, adopted the definition from the US National Coalition for Cancer Survivorship (NCCS), which defines a cancer survivor as anyone ‘living with, through, and beyond a cancer diagnosis’, encompassing the entire cancer continuum.

The Africa Survivorship Working Group, a multi-stakeholder special interest group associated with the Africa Cancer Research and Control ECHO [14], convened in late 2020 to develop plans for a 360° situational analysis of the current state of survivorship in Africa. The aim was twofold: (1) understand the patient and caregiver experience, the clinical perspective, and the policy gaps and opportunities; and (2) disseminate this information to help guide efforts to address gaps in care in the region. Within the scope of the Africa Survivorship Working Group situational analysis, Garton et al. [1] conducted a study examining survivorship care strategies outlined in NCCPs in Africa and found that while survivorship strategies were addressed in all 21 of the reviewed plans, more than half of the strategies focused primarily on palliative care, and few strategies included associated indicators. Another recent global review of NCCPs found a similar trend where most NCCPs from the region lacked measurable goals and objectives for survivorship care [13].

To complement the existing understanding of survivorship care strategies of African countries, and to deepen understanding about the scope of current practices in survivorship care, our study team within the Africa Survivorship Working Group sought to conduct a scoping review of post-treatment survivorship in Africa, taking into consideration the diversity of contextual environments in the region, and the possibility to identify and share innovative locally driven practices. A scoping review allows for mapping and identifying gaps in the literature on a given topic which can be used for decision-making and further research [15]. Our review presents findings from a review of current survivorship practices in Africa, offering insights into key areas of focus, existing strengths, and gaps that need to be addressed. For this scoping review, the focus was on the post-treatment survivorship phase and its associated factors.

## Methods

### Protocol and registration

The scoping review methods from the Joanna Briggs Institute were followed for the conduct of this review [16]. A protocol was written a priori following the Preferred Reporting Items for Systematic Reviews and Meta Analyses extension for Scoping Reviews (PRISMA-ScR) checklist [17]. The protocol was registered on the Open Science Framework. The PRISMA-ScR was used for reporting of this review.

### Eligibility criteria

The study selection process adhered to the following criteria. Studies published in English, from January 2011 to August 2024, involving either adult or paediatric oncology populations were considered. Accepted article types included primary research, conference abstracts, and programme descriptions. The eligible study designs encompassed case reports, case series, cross-sectional surveys, case–control studies, cohort studies, randomised controlled trials, focus group discussions, key informant interviews, and ethnographic research.

Additional inclusion criteria were:

- Inclusion of records if they focused on survivors and/or discussed survivorship care and related factors. For this study, survivorship care referred to the follow-up care provided to patients diagnosed with any cancer at any age after the completion of their primary cancer treatment.
- Inclusion of studies conducted within any of the 54 nations of Africa, as defined by the United Nations.
- Inclusion of studies even if they represented multiple countries, including at least one African country, with separate reporting of African participant/survivor data.
- Inclusion of studies documenting post-treatment information (at least 3 months after treatment completion) up to, but not including end-of-life care.
- Inclusion of study populations with metastatic or advanced disease.
- Inclusion of studies on patients 1-year post-diagnosis who are no longer undergoing primary treatment unless the article specifies ongoing primary treatment.
- Inclusion of studies focusing on survivorship care, such as treatment summaries and care plans, survivorship-related factors, such as financial toxicity, re-entering the workforce, late- or long-term side effects from cancer or its treatment (including physical, psychological, emotional, spiritual, and social aspects), stigma, patient navigation, access to primary care, family or spousal relationships, cancer recurrence screening for subsequent malignancies, lifestyle and behaviour change for cancer prevention, patient and family education, primary care utilisation and care coordination, rehabilitation, and quality of life.
- Inclusion of studies of survivors on long-term therapy like hormonal therapy or Imatinib (Gleevec, a tyrosine kinase inhibitor).
- Inclusion of studies focusing on healthcare providers of survivorship care, family or caregivers of survivors, lived experiences of cancer survivors post-treatment, and palliative care that is not end-of-life.
- Inclusion of studies where survivors are involved in the intervention (e.g. survivors participating in community education on cancer awareness or leading support groups for patients undergoing treatment).
- Inclusion of longitudinal studies that evaluate survivors after completion of their primary cancer treatment.

Studies were excluded if the study was not published in English or no translation existed, if not conducted in Africa, if survivorship care was focused on the period of diagnosis or primary treatment, if the study reported on immigrants from LMICs who are now receiving survivorship services in high-income countries (HICs) and if end-of-life care was the focus of the study. Other exclusion criteria were if the study was on length of survival or treatment after cancer recurrence, if the study type was a review article, commentary article, if the study design was a clinical trial or pertained to tool validity studies, or where survivors were only mentioned in the discussion section relating to implications for survivorship but where the study itself was not focused on survivors or survivorship.

### Information sources and search

The following databases were searched by a biomedical librarian (AAL) in August 2021, January 2023, and August 2024: Cumulative Index to Nursing and Allied Health Literature Plus (CINAHL Plus) (EBSCOhost), Embase (Elsevier), PubMed (US National Library of Medicine), and the Web of Science: Core Collection (Clarivate Analytics). The search strategies were developed by the biomedical librarian with feedback from the review team. The searches were comprised of keywords and controlled vocabulary terms (e.g. CINAHL Subject Headings [CINAHL Plus], EMTREE [Embase], MeSH [PubMed]) for each concept of interest (i.e. cancer, survivorship/survivors, Africa) (see Online Resource 1 for final search strategies used). The search parameters were limited by publication year (2011–August 2024) and language (English). We specifically chose 2011 to offer a comprehensive 10-year perspective and focused on English due to language constraints and translation limitations within the team. Moreover, a search strategy was implemented to narrow the searches to include studies conducted in Africa, which aligns with our primary study location of interest. However, we acknowledge that some authors may not specify the geographical study setting in their titles or abstracts, potentially resulting in missed articles during the search. A search strategy was applied to exclude review articles, as outlined in our exclusion criteria. EndNote 20 (Clarivate Analytics) was utilised to collect and identify duplicate and unique citations.

### Selection of sources of evidence

Covidence (Veritas Health Innovations) was used for study selection (i.e. screening). A two-level screening process was conducted. First the titles and abstracts of all unique records retrieved from the literature searches were screened, and then the full text screened of those records included after title and abstract screening. Two reviewers (AZ, IG, GN, MAR, MKC, NP, TO) independently and in duplicate screened each record at both levels. All discrepancies at both levels were resolved by a different reviewer (either MKC, GN, AZ, or NP). Prior to commencement, a pilot was conducted at the title and abstract level with all reviewers in Covidence on a sample of records selected by the librarian. After the pilot, the eligibility criteria were revised as necessary, and protocol updated.

### Data collection and data items

Data collection was completed in Covidence (Online Resource 2 for copy of data extraction form). Two reviewers (AZ, IG, GN, MAR, MKC, NP, TO) collected the data from each article independently and in duplicate, and discrepancies in the collected data were resolved by a different reviewer (AZ, GN, MKC, NP) using the consensus feature in Covidence. During data collection, missing or unclear data were marked as missing, and discussed with the team to determine how to resolve the issues. If during data collection, it was determined that a conference abstract and full journal article was included and that they may be reporting on the same study and/or results, the two records were compared by reviewing the title, authors, and text. If the two records reported the same study, the records were merged in Covidence, and the data collected once for that study (but still in duplicate by two reviewers). If the two records were not reporting the same study, each record was included and proceeded through data collection.

A pilot of the data collection process was completed twice in Microsoft Excel prior to starting using 10 articles for each set selected by the biomedical librarian. All reviewers participated in the pilots. Two pilots were completed to refine the data items to collect from each included article. After completing the pilot, the data collection form in Covidence was created to reflect the revisions and clarifications resulting from the pilot. For the pilot, Microsoft Excel was used so that reviewers collected the data from the same article thereby allowing for comparison between each reviewer’s data and to have the same collection experience.

The types of data extracted included study type, setting, target population, cancer type, geographic location, key groups involved, and long-term effects addressed. The domains of long-term effects included were derived from the National Academies of Science, Engineering and Medicine’s framework outlined in From Cancer Patient to Cancer Survivor: Lost in Transition [12], with a specific focus on physical effects, psychosocial effects, care coordination, and quality of life [18]. These categories were selected to create a descriptive analysis, and to identify characteristics of current practices.

### Synthesis of results

The final consensus data with discrepancies resolved was exported from Covidence into Microsoft Excel for further cleaning and analyses. Two reviewers completed data cleaning and analyses (AZ, MAR), and data were further categorised and standardised to allow for analysis. Google Sheets was used for creating the data dictionary for data cleaning and analyses (see Online Resource 3). Results are presented in a narrative summary with descriptive statistics and a table of key study characteristics of the included studies in addition to tables and figures displaying specific findings of interest.

## Results

**Fig. 1:**
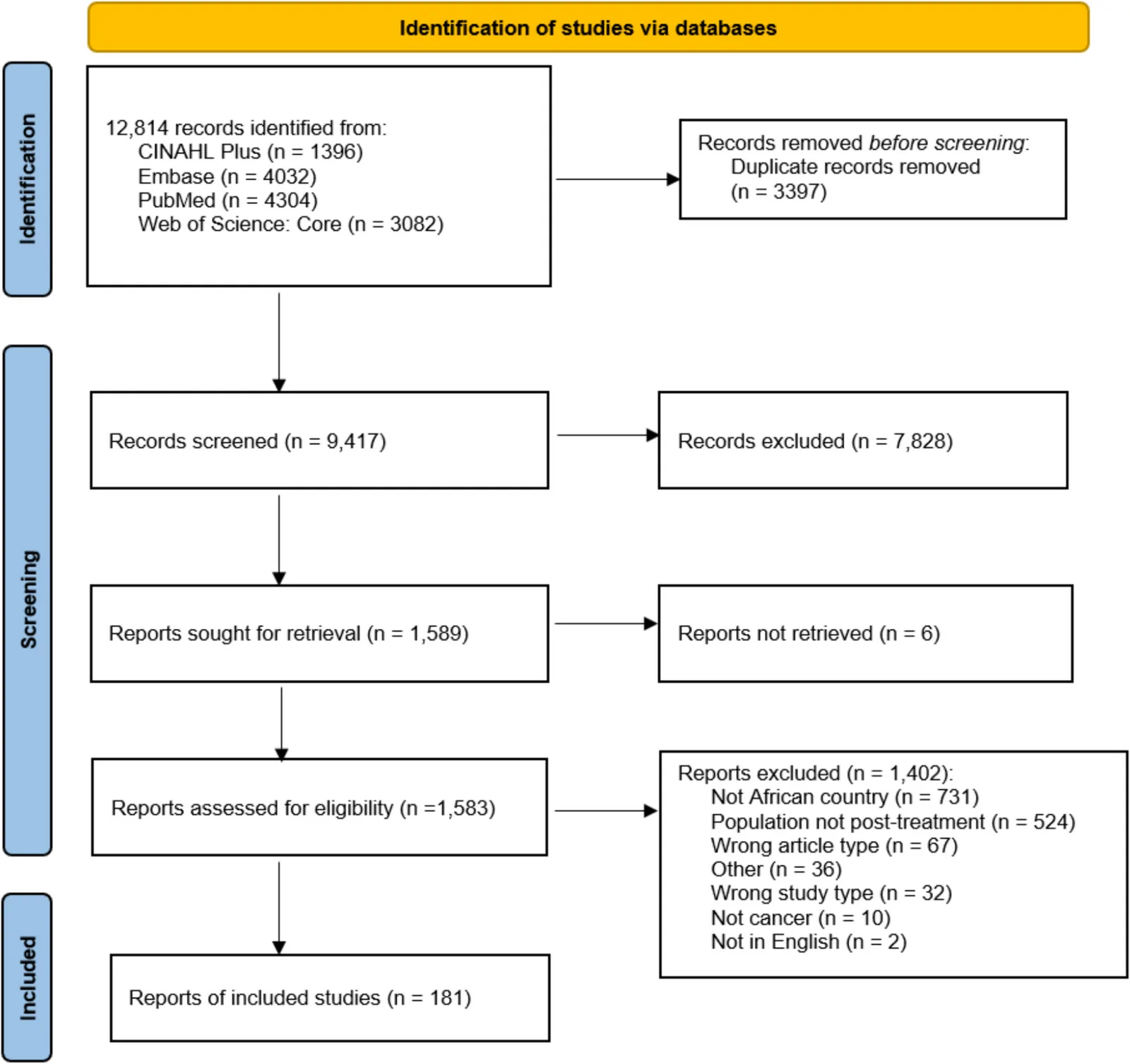
PRISMA flow diagram of study selection process

The searches identified 12,814 records from four databases. After removing duplicates, the titles and abstracts of 9417 records were screened. Of these, 1589 were included and passed to full-text screening. After full-text screening, 1408 were excluded and 181 articles included (Fig. 1).

### Characteristics of included studies

Of the 181 included studies, 110 (61%) were journal articles, and 71 (39%) conference abstracts. Online Resource 4 shows studies by publication year. There was a steady upward trend showing an increase in publications focused on post-treatment survivorship, from six studies in 2011 to 22 through August 2024 (with a peak of 32 studies in 2022 and 2023). With regard to study type, 97(54%) were quantitative, 42 (23%) were qualitative, 25 (14%) were programme descriptions, and 18 (10%) employed mixed methods. Individual studies had different settings. Most commonly, 92 (51%) of the studies were reported from a hospital or clinic, 38(21%) from a cancer centre, 29 (16%) were community based, and the remainder 15 (8%) were done in other settings (e.g. social media, other types of healthcare facilities, universities, and conferences) or unspecified (Online Resource 5).

**Fig. 2:**
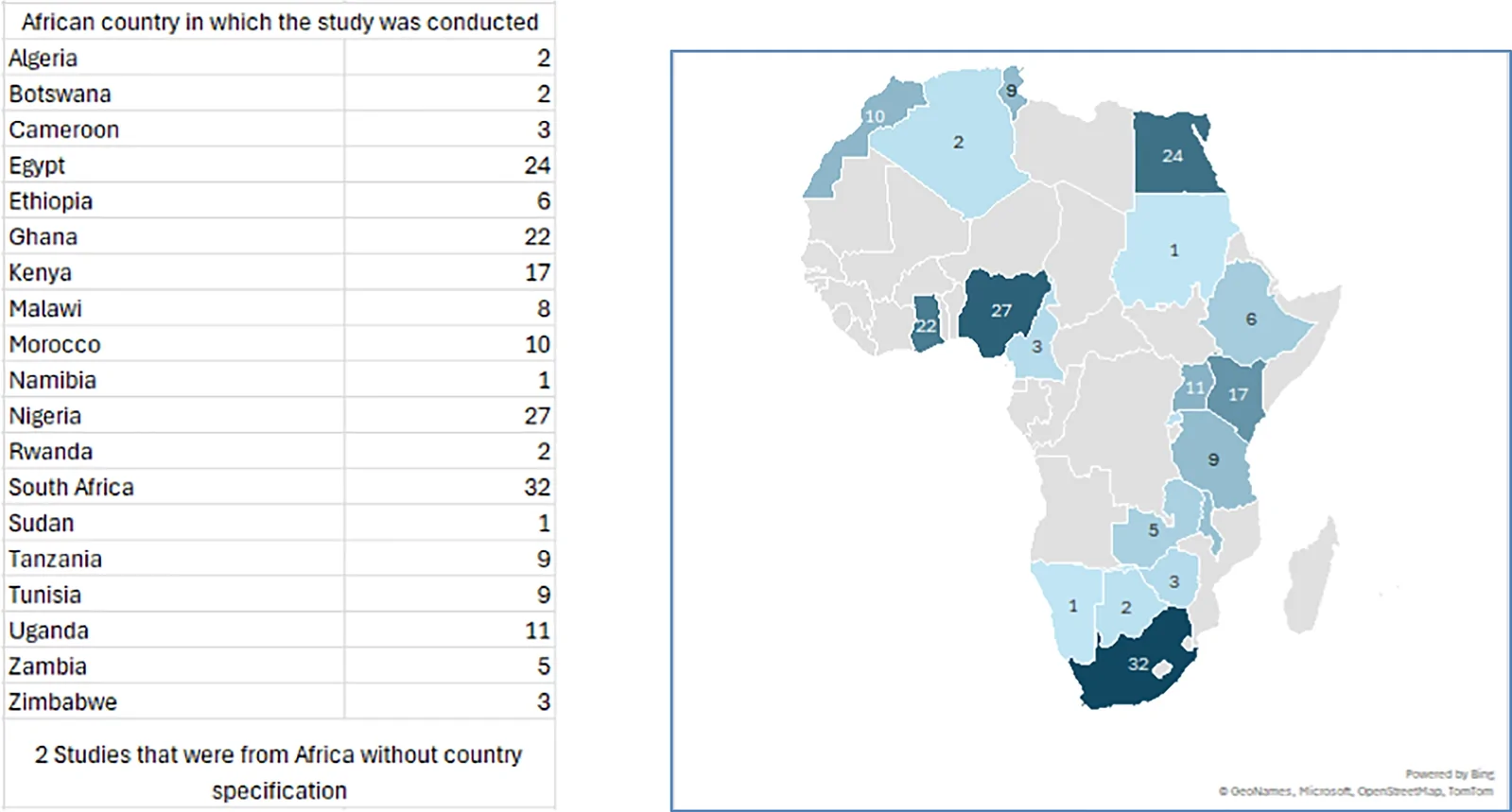
Geographical distribution of included studies. Note: Multiple countries may be the focus of a single study

In terms of geographical distribution, this scoping review encompassed data from 19 of the 54 countries in the African continent. Figure 2 displays the cumulative number of studies conducted in each country; the number exceeds 181 as some studies were conducted in multiple countries. Additionally, two studies lacked specific country identifiers and were reported as originating from Africa. South Africa emerged as the most frequently represented country with a total of 32 studies, accounting for 18% of the included research. Nigeria followed closely with 27 studies (15%) and Egypt with 24 studies (13%). Ghana and Kenya were also highly represented with 22 (12%) and 17 (9%) respectively.. Breaking down the distribution by region, 46 studies took place in North Africa, comprising Algeria, Egypt, Morocco, Sudan, and Tunisia. West Africa was represented by Nigeria and Ghana, which had 49 studies. Central Africa’s presence in the review was primarily through Cameroon, contributing three studies. East Africa, on the other hand, included studies from eight countries, including Ethiopia, Kenya, Malawi, Rwanda, Tanzania, Uganda, Zambia, and Zimbabwe, for a cumulative total of 61 studies. Finally, Southern Africa was represented by Botswana, Namibia, and South Africa, which together had 35 studies included in the review.

Most of the 181 studies primarily centred on one survivorship-related population. However, a significant portion included multiple study populations, such as survivors and healthcare workers, as well as survivors and caregivers. Survivors represented the most prominent study population, encompassing 89% of the studies. Oncology healthcare workers and caregivers each comprised study populations in 23% of the research. Some studies also featured other populations, including community healthcare workers and patient navigators (3%), non-oncology healthcare workers (3%), advocates (3%), and community leaders and policymakers (3%).

The word ‘survivor(-ship)’ appeared in 154/181 (85%) of the studies; however, a definition of survivorship was present only in 9/154 (10%) of those studies. Just 40 (22%) of the studies focused on survivors of childhood cancer. While 75% of childhood cancer studies investigated survivorship in the context of paediatric cancers as a collective entity, without specifying or combining multiple cancer types, the remaining 25% were dedicated to survivorship following specific diagnosis. This subgroup included three studies on survivorship after paediatric leukaemia, three studies on paediatric lymphoma survivorship, one study on hematologic malignancies (joining both leukaemias and lymphomas), two studies on retinoblastoma survivorship (jointly with osteosarcoma in one of these studies), and one study on paediatric medulloblastoma (a brain cancer) survivorship.

Although most studies concentrated on survivorship of individual cancer types, many involved a diverse patient population. Breast cancer was mentioned in 51% of studies, followed by hematologic malignancies (leukaemia, lymphoma, and myeloma) in 28% of studies. Genitourinary cancers (excluding cervical cancer) were referenced in 12% of the studies, while unspecified childhood cancer was mentioned in a close percentage of 11% of the studies. Also, just 6% of the studies mentioned post-treatment surveillance for recurrent or subsequent cancers. For a full description of mentioned cancer types by number of studies, see Online Resource 6.

Long- or late-term physical effects of cancer and its treatment were mentioned in 56% of studies. Most common effects mentioned were sexual health (22%), pain (19%), gastrointestinal symptoms (15%), and fatigue (13%). Additional physical effects mentioned were weight loss (11%), neurologic symptoms (9%), and sleep difficulties (8%). Least reported was infections (1%). For full details on physical effects of cancer and its treatment, see Table 1.

**Table 1:** Physical and psychosocial effects of cancer and its treatment

	Number of studies	Percentage (%)
Physical effects		
Sexual health	40	22.1%
Pain	34	18.8%
GI symptoms	27	14.9%
Fatigue	24	13.3%
Weight loss	19	10.5%
Infertility	18	9.9%
Neurological symptoms	16	8.8%
Sleeping difficulty	15	8.3%
Lymphedema	13	7.2%
Musculoskeletal problems	10	5.5%
Metabolic problems/obesity	10	5.5%
Lung toxicity	9	5.0%
Cardiotoxicity	9	5.0%
Other	8	4.4%
Hair loss	5	2.8%
Urinary symptoms	4	2.2%
Assistive equipment	4	2.2%
Infections	2	1.1%
Psychosocial effects		
Medical debt/financial toxicity	43	23.8%
Anxiety	41	22.7%
Stigma	38	21.0%
Body image alterations	36	19.9%
Depression	35	19.3%
Fear of death	25	13.8%
Community rejection	18	9.9%
Return to work difficulty	15	8.3%
Other	8	4.4%
Spousal abandonment	8	4.4%
Psychological concerns (unspecified)	6	3.3%
Family conflict	4	2.2%
School/education difficulties	3	1.7%
Post-traumatic growth	2	1.1%

Physical Effects: Multiple physical effects may be mentioned in a single study. *GI*, gastrointestinal; Other: *GVHD*, gynaecological symptoms, hepatic dysfunction, hypothalamic pituitary deficiency, physical deformities, radiation-induced fibrosis, skin discoloration

Psychosocial Effects: Multiple psychosocial effects may be mentioned in a single study. Other: compassion fatigue, disturbed peer relationships, functionality, healthcare system difficulties, lack of coping, masculine identity problems, spiritual wellbeing

Psychosocial effects of cancer and its treatment were mentioned in 57% of all studies. Most common effects mentioned were medical debt and financial toxicity (24%), anxiety (23%), stigma (21%), and body image alterations (19%). For full details on psychosocial effects of cancer and its treatment, see Table 1. Care coordination between different stakeholders was mentioned in 12% of all studies, largely between oncologists and other specialised healthcare practitioners (76%), but also between oncologists and primary care providers (24%), and between oncologists and community leaders (19%). Other types of care coordination were reported in one to two studies.

Key groups mentioned in relation to survivorship care delivery were caregivers (19%), peer survivors (13%), and nurses and primary or general practitioners (8% respectively). For full details, see Table 2. Out of 181 studies, only 32 (18%) featured an intervention component. These interventions encompassed various elements, with 12 (38%) involving education and training, 12 (38%) involving support or supportive care, five (15%) involving awareness and other programmes, and three (9%) involving other types of interventions. Quality of life (QoL) was measured via a questionnaire/survey in 31% of studies and was the primary purpose in 25%.

**Table 2:** Groups mentioned in survivorship care delivery studies

Key groups mentioned in studies related to survivorship care delivery	Number of studies	Percentage (%)
None	101	55.8%
Caregivers*	34	18.8%
Peer survivors**	23	12.7%
Nurses	14	7.7%
Primary care or general practitioners	14	7.7%
Policymakers	7	3.9%
Patient navigators	7	3.9%
Cancer centres	7	3.9%
Faith-based organisations	7	3.9%
NGOs	6	3.3%
Community healthcare workers	5	2.8%
Physiotherapists	3	1.7%
Home-based care providers	2	1.1%
Fertility specialists	2	1.1%
Clinical psychologists	1	0.6%
Designers	1	0.6%
Employers	1	0.6%
Stakeholders	1	0.6%
Teachers	1	0.6%

Multiple groups may be mentioned in a single study. *NGOs*, non-governmental organisations. *Caregivers included as follows: family, friends, community members who care for the survivors. **Peer survivors included as follows: survivor-led support groups, peer-to-peer support, survivor-led advocacy campaigns

## Discussion

This study presents the scope of research and reports on post-treatment survivorship in Africa in the past decade (2011–2024) and shows a significant upward trend in research output over that time. This suggests a growing attention to the post-treatment cancer survivorship domain in Africa, at least in the realm of scientific writing about survivorship care, care-related factors, and research. Coverage of only 19 of 54 African countries highlights a significant limitation in terms of geographical representation. This suggests that these findings may not be reflective of the full scope of post-treatment survivorship work in the continent. However, the limited availability of NCCPs from the region, as highlighted in studies by Manirakiza et al. [19] and Mullen et al. [13], mirrors the limited post-survivorship literature for a substantial number of countries. The observation that South Africa, Egypt, Nigeria, Kenya, and Ghana are leading in terms of research output might reflect the health infrastructure in place for cancer control, and possibly the level of research opportunities, and scientific writing outputs of these countries [20].

The term ‘survivorship’ appears in many of the included studies, suggesting that it is a widely recognised and discussed concept in the literature. However, only a small number of the included studies defined or described ‘survivorship’ or ‘survivor’. As reported in review by Marzorati et al. [21], and more recently from other survivorship-focused reviews in Africa and globally (Garton et al. [1] and Mullen et al. [13] respectively), this lack of consistency and clarity regarding the concept may potentially lead to varying interpretations and approaches to post-treatment survivorship care. The significant gap between the use of the term and its description highlights the need for consensus and greater clarity for research and practice, as the understanding of components to include in the survivorship domain continues to expand [22, 23]. Establishing a universally acceptable definition of survivorship and survivorship related factors could enhance communication, research comparability, and the development of more effective post-treatment cancer care strategies.

A majority of studies were carried out in hospital settings and cancer centres, with only a few were situated in community settings and university-affiliated hospitals. Meanwhile, key participants in many of the studies were fellow caregivers and peer survivors. This creates a notable discrepancy between the reported settings in the literature and the places where actual care is taking place and those involved in care delivery. With most studies conducted in cancer centres, this potentially overlooks significant work occurring in community settings, where survivors, their peers, and caregivers reside. The result is a possible underreporting of community-based survivorship initiatives, contrasting with survivorship activities in the clinical settings that dominate published research. There is a need for increased support for community-based participatory research and opportunities for peer survivors and caregiver voices to be represented in the literature.

There was a balanced mix of study designs including quantitative, qualitative, programme descriptions, and mixed methods; most of the studies were observational, with few interventional studies or randomised trials. While observational study designs offer advantages in terms of cost-effectiveness and efficiency for examining variable associations, they are limited in establishing causation or predictive relationships [24]. More causality-determining or experimental methodologies, particularly in understanding context-specific survivorship issues such as stigma, are needed. Increased emphasis on interventional studies, perhaps through targeted funding, is crucial for the development and delivery of comprehensive, evidence-based survivorship care.

This scoping review identified a wide array of cancer types, with breast and hematologic cancers being the most widely studied. The focus on breast cancer could be due to the increased awareness, advocacy, and dedicated funding of breast cancer research [25]. Paediatric cancers, despite their unique challenges [26], were underrepresented with only 22% focusing on this population There may be a variety of explanations for this, thus making it difficult to collect substantial amounts of data which are representative of post-treatment survivorship care. Also, many paediatric cancer patients are diagnosed at a very young age and may not reach the age for which long-term survivorship care is provided, especially with the current lack of access to essential paediatric cancer medicines as recently reported by Petrrica et al. [27]. Therefore, there is a need for increased attention towards this population considering their unique needs and challenges, especially in the post-treatment phase.

Both psychosocial and physical effects of cancer and its treatment were equally reported in the studies included in this review. That financial toxicity and medical debt was reported as the major psychosocial concerns, points towards the economic challenges faced by cancer survivors in the African region, which is a shared challenge globally [28]. This aligns with the findings of Garton et al. [1] where none of the reviewed NCCPs in Africa included an available/affordable insurance strategy. Moreover, stigma, either direct or associated with spousal abandonment and community rejection, was frequently noted in the studies, suggesting a need for research to examining the underlying factors and mechanisms of stigma and evaluate for potential contextually relevant interventions. In terms of physical effects, sexual health issues, pain, and fatigue were predominantly reported. The findings are consistent with a recent review of publications in the *Journal of Cancer Survivorship* [29] and an analysis of abstracts presented at the International Psycho-Oncology Society (IPOS) World Congress meetings [30] where similar issues were identified as predominant in survivorship research. This underscores the ongoing necessity for concerted efforts to address these challenges.

Only a small number of studies reported care coordination, which is crucial for holistic survivorship care, from a multidisciplinary point of view [31]. Where reported, most of this coordination was between oncologists and specialised healthcare practitioners. Moving forward, it would be important to study the current and potential involvement of all the stakeholders in the care coordination process, including the survivors, their caregivers, and community resources. This approach has been associated with improved patient outcomes, reduced costs, and patient and healthcare satisfaction [31]. The mention of community leaders suggests an understanding of the socio-cultural dimensions of survivorship care within the African region. The reality is that there is likely a transition of survivorship care from specialised cancer centres to the broader community, including individual caregivers, and this is not represented in the literature or in the current care models. Given Africa’s resource limitations and the possibility that this approach might be more effective, further research is needed to examine its effectiveness. Africa’s rich primary care workforce, such as community health promoters [32], could be trained to address certain aspects of survivorship care and survivorship-related factors. The World Health Organization (WHO) recognises the community health workforce as crucial assets in improving access to care [33, 34]. Such strategies have proven effective for other diseases, for instance in mental health via the mhGAP frameworks [35–37] and in HIV/AIDS and tuberculosis [38, 39]. Additionally, this approach could help in awareness raising, an essential domain in the NAM framework [12] and help mitigate the stigma that sometimes surrounds a cancer diagnosis in certain communities. Moreover, shared care models and primary care-led models have demonstrated effectiveness comparable to specialist-led models in the context of survivorship care and may be a feasible alternative in Africa [40, 41].

Only one-fifth of the studies discussed interventions, with education and training and support/supportive care being most common. This suggests a potential area that could be further explored and developed to improve survivorship outcomes, or a gap in the reporting on other interventions. This may suggest that research concerning other important aspects of post-treatment survivorship care, such as psychosocial support, lifestyle interventions, or innovative technologies, are not in use commonly. The popularity of support groups and educational materials may warrant further examination to examine if these interventions have demonstrated efficacy, are less costly, or are perceived as valuable. However, it is important for research to continue assessing and refining these interventions to ensure they are evidence-based and meet the evolving needs of survivors. As survivorship-related needs can vary significantly depending on the type of cancer, treatment, and individual characteristics, relying heavily on a few interventions may not adequately address the diverse needs of cancer survivors. Therefore, tailored interventions for specific populations, developed by and with input from survivors, are required [42, 43].

Finally, quality of life was only measured as a primary outcome in one-third of the studies. While this is encouraging considering that quality of life is the ultimate goal of survivorship interventions, more work is needed, particularly in underrepresented populations such as the paediatrics, adolescents, and young adults. Overall, more interventional studies and reporting on effective intervention strategies to address the diverse needs of cancer survivors are needed in Africa. Consequently, this would inform the development and delivery of context-specific and evidence-based survivorship care.

### Study limitations

The inconsistency and varying use of terms to describe ‘survivor’ and ‘survivorship’ created challenges when searching and screening as some relevant studies may have been missed or excluded. However, we tried to use a variety of terms and sources and sought input from experts in the field of survivorship to help define and interpret the terminology used for and in our review. Limiting to English language literature and not relying on free open-source translation tools, which although they are available cannot be considered official translations, is a limitation in this review. Additionally, there may be a lack of published literature as much survivorship care is happening at the community level and study settings identified in this review were largely at the facility level.

## Conclusions

Our scoping review highlights a growing body of research on post-treatment cancer survivorship care and related factors in Africa. However, the existing literature originates from only one-third of the continent’s countries, with most studies being facility-based. While substantial research has explored the physical and psychosocial effects of cancer, there is a pressing need for more studies on care coordination, community-driven research, targeted interventions, and quality-of-life outcomes. Financial toxicity and stigma emerge as significant challenges for survivors in the post-treatment phase. The predominance of studies on breast cancer survivors underscores both the depth of research in this area and the need to expand focus to other cancer types. Given Africa’s diverse socio-cultural and economic landscape, more country-specific research—particularly from underrepresented nations—is crucial for developing holistic and contextually relevant survivorship care strategies.

## Supplementary Information

Below is the link to the electronic supplementary material.

ESM 1 (PDF 129 KB)

ESM 2 (PDF 339 KB)

ESM 3 (PDF 142 KB)

ESM 4 (PDF 369 KB)

## Data Availability

No datasets were generated or analysed during the current study.
