# Supplementary material for: A scoping review exploring cancer survivorship in Africa: 2011 to 2024
Source: J Cancer Surviv. 2025 Apr 25;20(Suppl 1):1127–37. doi: 10.1007/s11764-025-01805-5 (PMC13676652; doi:10.1007/s11764-025-01805-5)
Supplement: Supplementary file 1 [file 11764_2025_1805_MOESM1_ESM.pdf]

Final Search Strategies

Title of Review: A scoping review exploring cancer survivorship care in Africa: 2011 to 2024

Database: PubMed/MEDLINE
Platform: US National Library of Medicine
Date Searched: August 3, 2021 / **Date Search Updated:** January 23, 2023 / **Date Search Updated:** August 5, 2024
Date Limits: Publication Year: 1/1/2011–12/31/2024
Other Limits/Filters: Language: English

Set	Concept	Search Strategy
#1	Cancer	(neoplasm*[Title/Abstract] OR cancer*[Title/Abstract] OR oncolog*[Title/Abstract] OR carcinoma*[Title/Abstract] OR sarcoma*[Title/Abstract] OR adenocarcinoma*[Title/Abstract] OR malignan*[Title/Abstract] OR neoplasia*[Title/Abstract] OR leukemia*[Title/Abstract] OR leukaemia*[Title/Abstract] OR lymphoma*[Title/Abstract] OR melanoma*[Title/Abstract] OR myeloma*[Title/Abstract] OR neoplasms[Mesh] OR “Leukemia”[Mesh] OR “Lymphoma”[Mesh] OR “Carcinoma”[Mesh] OR “Melanoma”[Mesh] OR “Sarcoma”[Mesh] OR “Adenocarcinoma”[Mesh])
#2	Survivorship	(Survivorship[Mesh] OR Aftercare[Mesh] OR “after care”[Title/Abstract] OR survivorship[Title/Abstract] OR aftercare[Title/Abstract] OR “followup care”[Title/Abstract] OR “follow up care”[Title/Abstract] OR “post treatment surveillance”[Title/Abstract] OR “posttreatment surveillance”[Title/Abstract] OR “post treatment care”[Title/Abstract] OR “posttreatment care”[Title/Abstract] OR “Cancer Survivors”[Mesh] OR “Cancer Survivor*”[Title/Abstract] OR “cancer recurrence*”[Title/Abstract] OR “Neoplasm Recurrence, Local/prevention and control”[Majr] OR “Neoplasm Recurrence, Local/secondary”[Majr] OR “longterm side effect*”[Title/Abstract] OR “long term side effect*”[Title/Abstract] OR “late term side effect*”[Title/Abstract] OR “late side effect*”[Title/Abstract] OR “Primary Health Care”[Majr] OR “primary health care”[Title/Abstract] OR “Continuity of Patient Care”[Majr] OR “continuity of care”[Title/Abstract] OR “care continuity”[Title/Abstract] OR “care continuum”[Title/Abstract] OR “primary care”[Title/Abstract] OR “Case Management”[Majr] OR “Patient Care Planning”[Mesh:NoExp] OR “patient care plan*”[Title/Abstract] OR “case management”[Title/Abstract] OR “care coordination”[Title/Abstract] OR “coordinating care”[Title/Abstract] OR “coordinated care”[Title/Abstract] OR “financial toxicit*”[Title/Abstract] OR bankruptc*[Title/Abstract] OR “financial stress*”[Title/Abstract] OR “financial distress”[Title/Abstract] OR “workforce reentry”[Title/Abstract] OR “workforce re entry”[Title/Abstract] OR “return to work”[Title/Abstract] OR “back to work”[Title/Abstract] OR “quality of

		life"[Title/Abstract] OR "family stress*"[Title/Abstract] OR "family relationship*"[Title/Abstract] OR "spousal relationship*"[Title/Abstract] OR "spouse relation*"[Title/Abstract] OR "lifestyle chang*"[Title/Abstract] OR "life style chang*"[Title/Abstract] OR "lifestyle modification*"[Title/Abstract] OR "life style modification*"[Title/Abstract] OR "behavior chang*"[Title/Abstract] OR "behaviour chang*"[Title/Abstract] OR "changing behavior*"[Title/Abstract] OR "changing behaviour*"[Title/Abstract] OR stigma[Title/Abstract] OR "patient navigation"[Title/Abstract] OR "patient navigator"[Title/Abstract] OR "Patient Navigation"[Majr] OR "Social Stigma"[Majr] OR "Return to Work"[Majr] OR "Quality of Life"[Majr])
#3	Geographical Focus	("Developing Countries"[Mesh] OR "low resource"[Title/Abstract] OR "developing country"[Title/Abstract] OR "developing countries"[Title/Abstract] OR LMIC[Title/Abstract] OR LMICs[Title/Abstract] OR "lower middle income"[Title/Abstract] OR "lmics"[Title/Abstract] OR "lower middle income"[Title/Abstract] OR "lower middle income countries"[Title/Abstract] OR "lower middle income country"[Title/Abstract] OR "lower middle income economies"[Title/Abstract] OR "lower middle income setting"[Title/Abstract] OR "lower middle income settings"[Title/Abstract] OR "low and middle income countries"[Title/Abstract] OR "low- and middle- income country"[Title/Abstract] OR "low and middle income"[Title/Abstract] OR "low- and middle income"[Title/Abstract] OR "developing nation"[Title/Abstract] OR "developing nations"[Title/Abstract] OR "developing population"[Title/Abstract] OR "developing populations"[Title/Abstract] OR "developing world"[Title/Abstract] OR "less developed country"[Title/Abstract] OR "less developed countries"[Title/Abstract] OR "less developed nation"[Title/Abstract] OR "less developed nations"[Title/Abstract] OR "less developed world"[Title/Abstract] OR "lesser developed countries"[Title/Abstract] OR "lesser developed nations"[Title/Abstract] OR "under developed country"[Title/Abstract] OR "under developed countries"[Title/Abstract] OR "under developed nations"[Title/Abstract] OR "under developed world"[Title/Abstract] OR "underdeveloped country"[Title/Abstract] OR "underdeveloped countries"[Title/Abstract] OR "underdeveloped nation"[Title/Abstract] OR "underdeveloped nations"[Title/Abstract] OR "underdeveloped population"[Title/Abstract] OR "underdeveloped populations"[Title/Abstract] OR "underdeveloped world"[Title/Abstract] OR "middle income country"[Title/Abstract] OR "middle income countries"[Title/Abstract] OR "middle income nation"[Title/Abstract] OR "middle income nations"[Title/Abstract] OR "middle income population"[Title/Abstract] OR "middle income populations"[Title/Abstract] OR "low income country"[Title/Abstract] OR "low income countries"[Title/Abstract] OR "low income nation"[Title/Abstract] OR "low income nations"[Title/Abstract] OR "low income population"[Title/Abstract] OR "low income populations"[Title/Abstract] OR "lower income country"[Title/Abstract] OR "lower income countries"[Title/Abstract] OR "lower

		income nations"[Title/Abstract] OR "lower income population"[Title/Abstract] OR "lower income populations"[Title/Abstract] OR "underserved countries"[Title/Abstract] OR "underserved nations"[Title/Abstract] OR "poor country"[Title/Abstract] OR "poor countries"[Title/Abstract] OR "poor nation"[Title/Abstract] OR "poor nations"[Title/Abstract] OR "poor population"[Title/Abstract] OR "poor populations"[Title/Abstract] OR "poor world"[Title/Abstract] OR "poorer countries"[Title/Abstract] OR "poorer nations"[Title/Abstract] OR "poorer population"[Title/Abstract] OR "poorer populations"[Title/Abstract] OR "developing economy"[Title/Abstract] OR "developing economies"[Title/Abstract] OR "less developed economy"[Title/Abstract] OR "less developed economies"[Title/Abstract] OR "underdeveloped economies"[Title/Abstract] OR "middle income economy"[Title/Abstract] OR "middle income economies"[Title/Abstract] OR "low income economy"[Title/Abstract] OR "low income economies"[Title/Abstract] OR "lower income economies"[Title/Abstract] OR "low gdp"[Title/Abstract] OR "low gnp"[Title/Abstract] OR "low gross domestic"[Title/Abstract] OR "low gross national"[Title/Abstract] OR "lower gdp"[Title/Abstract] OR "lower gross domestic"[Title/Abstract] OR lmic[Title/Abstract] OR lmics[Title/Abstract] OR "third world"[Title/Abstract] OR "lami country"[Title/Abstract] OR "lami countries"[Title/Abstract] OR "transitional country"[Title/Abstract] OR "transitional countries"[Title/Abstract] OR "sub Saharan Africa"[Title/Abstract] OR "sub-saharan Africa"[Title/Abstract] OR "Africa South of the Sahara"[Mesh] OR "sub Saharan Africa"[Title/Abstract] OR "sub-saharan Africa"[Title/Abstract] OR "Africa South of the Sahara"[Mesh] OR "Africa, Central"[Mesh] OR "central Africa"[Title/Abstract] OR "East Africa" [Title/Abstract] OR "Eastern Africa"[Title/Abstract] OR "Africa, Eastern"[Mesh] OR "Southern Africa"[Title/Abstract] OR "Africa, Southern"[Mesh] OR "Western Africa"[Title/Abstract] OR "Africa, Western"[Mesh] OR "West Africa"[Title/Abstract] OR "north Africa"[Title/Abstract] OR "Northern Africa"[Title/Abstract] OR "Africa, Northern"[Mesh] OR "Asia, Southeastern"[Mesh] OR "Asia, Western"[Mesh] OR "West Asia"[Title/Abstract] OR "South East Asia"[Title/Abstract] OR "southeast asia"[Title/Abstract] OR "southeastern asia"[Title/Abstract] OR "South America"[Title/Abstract] OR "Latin America"[Title/Abstract] OR "Central America"[Title/Abstract] OR Africa[Title/Abstract] OR Asia[Title/Abstract] OR Caribbean[Title/Abstract] OR "West Indies"[Title/Abstract] OR "Central America"[Mesh:NoExp] OR "Latin America"[Mesh] OR "South America"[Mesh:NoExp] OR Afghanistan[Title/Abstract] OR Afghanistan[Mesh] OR Algeria [Title/Abstract] OR Algeria [Mesh] OR Belize[Mesh] OR Belize[Title/Abstract] OR Benin[Title/Abstract] OR Benin[Mesh] OR Botswana[Title/Abstract] OR Botswana [Mesh] OR "Burkina Faso"[Title/Abstract] OR "Burkina Fasso"[Title/Abstract] OR "Upper Volta"[Title/Abstract] OR "Burkina Faso"[Mesh] OR Burundi[Mesh] OR Burundi[Title/Abstract] OR

		Urundi[Title/Abstract] OR “Central African Republic”[Title/Abstract] OR “Central African Republic”[Mesh] OR Chad[Mesh] OR Chad[Title/Abstract] OR Comoros[Title/Abstract] OR Comoro Islands[Title/Abstract] OR Comores[Title/Abstract] OR Mayotte[Title/Abstract] OR “Democratic Republic of the Congo”[Mesh] OR Eritrea[Title/Abstract] OR Eritrea[Mesh] OR Eswatini [Title/Abstract] OR Eswatini[Mesh] OR Ethiopia[Title/Abstract] OR Ethiopia[Mesh] OR Gambia[Title/Abstract] OR Gambia[Mesh] OR Guinea[Title/Abstract] OR Guinea[Mesh] OR “Guinea-Bissau”[Title/Abstract] OR “Guinea-Bissau”[Mesh] OR Haiti[Title/Abstract] OR Haiti[Mesh] OR “North Korea”[Title/Abstract] OR “Democratic People's Republic of Korea”[Mesh] OR “Democratic People's Republic of Korea”[Title/Abstract] OR Liberia[Title/Abstract] OR Liberia[Mesh] OR Libya[Title/Abstract] OR Libya[Mesh] OR Madagascar[Title/Abstract] OR Madagascar[Mesh] OR Malawi[Title/Abstract] OR Malawi[Mesh] OR Nyasaland[Title/Abstract] OR Mali[Title/Abstract] OR Mali[Mesh] OR Mozambique[Title/Abstract] OR Mozambique[Mesh] OR Nepal[Title/Abstract] OR Nepal[Mesh] OR Niger[Title/Abstract] OR Niger[Mesh] OR Rwanda[Title/Abstract] OR Rwanda[Mesh] OR Senegal[Title/Abstract] OR Senegal[Mesh] OR Seychelles[Title/Abstract] OR Seychelles[Mesh] OR “Sierra Leone”[Title/Abstract] OR “Sierra Leone”[Mesh] OR Somalia[Title/Abstract] OR “Somalia”[Mesh] OR “South Africa”[Title/Abstract] OR “South Africa”[Mesh] OR “South Sudan”[Title/Abstract] OR “South Sudan”[Mesh] OR Sudan[Mesh] OR Sudan[Title/Abstract] OR Tanzania[Title/Abstract] OR Tanzania[Mesh] OR Togo[Mesh] OR Togo[Title/Abstract] OR “Togolese Republic”[Title/Abstract] OR Uganda[Title/Abstract] OR Uganda[Mesh] OR Zimbabwe[Mesh] OR Zimbabwe[Title/Abstract] OR Rhodesia[Title/Abstract] OR Angola[Mesh] OR Angola[Title/Abstract] OR Bangladesh[Mesh] OR Bangladesh[Title/Abstract] OR Bhutan[Mesh] OR Bhutan[Title/Abstract] OR Bolivia[Mesh] OR Bolivia[Title/Abstract] OR “cabo verde”[Title/Abstract] OR “cape verde”[Title/Abstract] OR “Cabo Verde”[Mesh] OR Cambodia[Mesh] OR Cambodia[Title/Abstract] OR Khmer Republic[Title/Abstract] OR Kampuchea[Title/Abstract] OR Cameroon[Title/Abstract] OR Cameroons[Title/Abstract] OR Cameron[Title/Abstract] OR “Cameroon”[Mesh] OR Congo[Title/Abstract] OR Zaire[Title/Abstract] OR Congo[Mesh] OR “Cote d'Ivoire”[Title/Abstract] OR “Ivory Coast”[Title/Abstract] OR “Cote d'Ivoire”[Mesh] OR Djibouti[Mesh] OR Djibouti[Title/Abstract] OR “French Somaliland”[Title/Abstract] OR Egypt[Title/Abstract] OR “United Arab Republic”[Title/Abstract] OR Egypt[Mesh] OR “el salvador”[Title/Abstract] OR “el salvador”[Mesh] OR “Equatorial Guinea”[Title/Abstract] OR “Equatorial Guinea”[Mesh] OR Gabon[Title/Abstract] OR Gabon[Mesh] OR Ghana[Title/Abstract] OR Ghana[Mesh] OR “Gold Coast”[Title/Abstract] OR Honduras[Title/Abstract] OR Honduras[Mesh] OR India[Mesh] OR India[Title/Abstract] OR Kashmir[Title/Abstract] OR Indonesia[Title/Abstract] OR Indonesia[Mesh] OR Iran[Title/Abstract] OR Iran[Mesh] OR

		Kenya[Title/Abstract] OR Kenya[Mesh] OR Kiribati[Title/Abstract] OR Kyrgyzstan[Mesh] OR “Kyrgyz republic”[Title/Abstract] OR Kyrgyzstan[Title/Abstract] OR Kirghizia[Title/Abstract] OR Kirghiz[Title/Abstract] OR Kirgizstan[Title/Abstract] OR Laos[Title/Abstract] OR “Lao PDR”[Title/Abstract] OR Laos[Mesh] OR Lesotho[Title/Abstract] OR Lesotho[Mesh] OR Mauritania[Title/Abstract] OR Mauritania[Mesh] OR Mauritius[Title/Abstract] OR Mauritius[Mesh] OR Micronesia[Title/Abstract] OR “Micronesia”[Mesh] OR Mongolia[Title/Abstract] OR Mongolia[Mesh] OR Morocco[Title/Abstract] OR Ifni[Title/Abstract] OR Morocco[Mesh] OR Myanmar[Title/Abstract] OR Burma[Title/Abstract] OR “Myanmar”[Mesh] OR Namibia[Title/Abstract] OR Namibia[Mesh] OR Nicaragua[Title/Abstract] OR Nicaragua[Mesh] OR Nigeria[Mesh] OR Nigeria[Title/Abstract] OR Pakistan[Title/Abstract] OR Pakistan[Mesh] OR “Papua New Guinea”[Title/Abstract] OR “Papua New Guinea”[Mesh] OR Philippines[Title/Abstract] OR Philipines[Title/Abstract] OR Phillipines[Title/Abstract] OR Phillippines[Title/Abstract] OR Philippines[Mesh] OR Samoa[Title/Abstract] OR Samoa[Mesh] OR “Sao Tome”[Title/Abstract] OR “Sao Tome and Principe”[Title/Abstract] OR “Sao Tome and Principe”[Mesh] OR “Atlantic Islands”[Mesh:NoExp] OR “Solomon Islands”[Title/Abstract] OR “Melanesia”[Mesh:NoExp] OR Melanesia[Title/Abstract] OR “Sri Lanka”[Title/Abstract] OR Ceylon[Title/Abstract] OR “sri lanka”[Mesh] OR Swaziland[Title/Abstract] OR Swaziland[Mesh] OR “Eswatini”[Mesh] OR Eswatini[Title/Abstract] OR Syria[Title/Abstract] OR Syria[Mesh] OR “Syrian Arab Republic”[Title/Abstract] OR Tajikistan[Title/Abstract] OR “Tajikistan”[Mesh] OR Tadzhikistan[Title/Abstract] OR Tadjikistan[Title/Abstract] OR Tadzhik[Title/Abstract] OR “East Timor”[Title/Abstract] OR “Timor Leste”[Title/Abstract] OR “Timor-Leste”[Mesh] OR Tunisia[Title/Abstract] OR Tunisia[Mesh] OR Ukraine[Title/Abstract] OR Ukraine[Mesh] OR Uzbekistan[Title/Abstract] OR “Uzbekistan”[Mesh] OR Uzbek[Title/Abstract] OR Vanuatu[Title/Abstract] OR Vanuatu[Mesh] OR “New Hebrides”[Title/Abstract] OR Vietnam[Title/Abstract] OR “Viet Nam”[Title/Abstract] OR “Vietnam”[Mesh] OR “West Bank”[Title/Abstract] OR “Gaza Strip”[Title/Abstract] OR Gaza[Title/Abstract] OR Yemen[Title/Abstract] OR “Yemen”[Mesh] OR Sanaa[Title/Abstract] OR Zambia[Title/Abstract] OR Zambia[Mesh])
#4		#1 AND #2 AND #3
#5	Limit: Publication Year & Language	#4 AND English[Language] AND ((“2011/01/01”[Date - Publication] : “2024/12/31”[Date - Publication]))

#6	Limit: Publication Type	#5 NOT (“systematic review*”[Title] OR “meta-analysis”[Publication Type] OR “meta-analysis”[Title] OR “meta-analyses”[Title] OR metanalyses[Title] OR metanalysis[Title] OR “meta analyses”[Title] OR “Review”[Publication Type] OR “Systematic Review”[Publication Type])

Notes: The limits for language (English) and publication year (2011–2024) were applied to the main search using the filters available in PubMed. The keywords were searched in the title and abstract fields in PubMed (i.e., [Title/Abstract]), title field (i.e., [Title]), and the controlled vocabulary terms are indicated with [Mesh] or as a Major MeSH term [Majr]. Phrases were enclosed in quotation marks to force the searching of the exact terms in order presented. To these results, the search strategy to exclude publication types not of interest as per eligibility criteria was used. No other limits were applied to the searches.

Database: Web of Science: Core Collection*
Platform: Clarivate Analytics
Date Searched: August 3, 2021 / **Date Search Updated:** January 23, 2023 / **Date Search Updated:** August 5, 2024
Date Limits: Publication Year: 2011–2024
Other Limits/Filters: Language: English; Document Type – exclude Review Article

Set	Concept	Search Strategy
#1	Cancer	TS=(neoplasm* OR cancer* OR oncolog* OR carcinoma* OR sarcoma* OR adenocarcinoma* OR malignan* OR neoplasia* OR leukemia* OR leukaemia* OR lymphoma* OR melanoma* OR myeloma*)
#2	Survivorship	TS=(survivorship OR aftercare OR "after care" OR "followup care" OR "follow up care" OR "post treatment surveillance" OR "posttreatment surveillance" OR "post treatment care" OR "posttreatment care" OR "Cancer Survivor*" OR "cancer recurrence*" OR "longterm side effect*" OR "long term side effect*" OR "late term side effect*" OR "late side effect*" OR "primary health care" OR "continuity of care" OR "care continuity" OR "care continuum" OR "primary care" OR "patient care plan*" OR "case management" OR "care coordination" OR "coordinating care" OR "coordinated care" OR "financial toxicit*" OR bankruptc* OR "financial stress*" OR "financial distress" OR "workforce reentry" OR "workforce re entry" OR "return to work" OR "back to work" OR "quality of life" OR "family stress*" OR "family relationship*" OR "spousal relationship*" OR "spouse relation*" OR "lifestyle chang*" OR "life style chang*" OR "lifestyle modification*" OR "life style modification*" OR "behavior chang*" OR "behaviour chang*" OR "changing behavior*" OR "changing behaviour*" OR stigma OR "patient navigation" OR "patient navigator")
#3	Geographical Focus	TS=("low resource" OR "developing country" OR "developing countries" OR LMIC OR LMICs OR "lower middle income" OR "lower middle income" OR "lower middle income countries" OR "lower middle income country" OR "lower middle income economies" OR "lower middle income setting" OR "lower middle income settings" OR "developing nation" OR "developing nations" OR "developing population" OR "developing populations" OR "developing world" OR "less developed country" OR "less developed countries" OR "less developed nation" OR "less developed nations" OR "less developed world" OR "lesser developed countries" OR "lesser developed nations" OR "under developed country" OR "under developed countries" OR "under developed nations" OR "under developed world" OR "underdeveloped country" OR "underdeveloped countries" OR "underdeveloped nation" OR "underdeveloped nations" OR "underdeveloped population" OR "underdeveloped populations" OR "underdeveloped world" OR "middle income country" OR "middle income countries" OR "middle income nation" OR "middle income nations" OR "middle income population" OR "middle income populations" OR "low income country" OR "low income countries" OR "low income nation" OR "low income nations" OR "low income population" OR "low income populations" OR "lower income

		country" OR "lower income countries" OR "lower income nations" OR "lower income population" OR "lower income populations" OR "underserved countries" OR "underserved nations" OR "poor country" OR "poor countries" OR "poor nation" OR "poor nations" OR "poor population" OR "poor populations" OR "poor world" OR "poorer countries" OR "poorer nations" OR "poorer population" OR "poorer populations" OR "developing economy" OR "developing economies" OR "less developed economy" OR "less developed economies" OR "underdeveloped economies" OR "middle income economy" OR "middle income economies" OR "low income economy" OR "low income economies" OR "lower income economies" OR "low gdp" OR "low gnp" OR "low gross domestic" OR "low gross national" OR "lower gdp" OR "lower gross domestic" OR lmic OR lmics OR "third world" OR "lami country" OR "lami countries" OR "transitional country" OR "transitional countries" OR "sub Saharan Africa" OR "sub-saharan Africa" OR "Africa South of the Sahara" OR "central Africa" OR "East Africa" OR "Eastern Africa" OR "Southern Africa" OR "Western Africa" OR "West Africa" OR "north Africa" OR "Northern Africa" OR "West Asia" OR "South East Asia" OR "southeast asia" OR "southeastern asia" OR "South America" OR "Latin America" OR "Central America" OR "West Indies" OR Afghanistan OR Algeria OR Belize OR Benin OR Botswana OR "Burkina Fasso" OR "Upper Volta" OR "Burkina Faso" OR Burundi OR Urundi OR "Central African Republic" OR Chad OR Comoros OR "Comoro Islands" OR Comores OR Mayotte OR "Democratic Republic of the Congo" OR Eritrea OR Eswatini OR Ethiopia OR Gambia OR Guinea OR "Guinea-Bissau" OR Haiti OR Libya OR "North Korea" OR "Democratic People's Republic of Korea" OR Liberia OR Madagascar OR Malawi OR Nyasaland OR Mali OR Mozambique OR Nepal OR Niger OR Rwanda OR Senegal OR Seychelles OR "Sierra Leone" OR Somalia OR "South Africa" OR "South Sudan" OR Sudan OR Sudan OR Tanzania OR Togo OR "Togolese Republic" OR Uganda OR Zimbabwe OR Rhodesia OR Angola OR Bangladesh OR Bhutan OR Bolivia OR "cabo verde" OR "cape verde" OR Cambodia OR "Khmer Republic" OR Kampuchea OR Cameroon OR Cameroons OR Cameron OR Zaire OR Congo OR "Cote d'Ivoire" OR "Ivory Coast" OR Djibouti OR "French Somaliland" OR Egypt OR "United Arab Republic" OR "el salvador" OR "Equatorial Guinea" OR Gabon OR Ghana OR "Gold Coast" OR Honduras OR India OR Kashmir OR Indonesia OR Iran OR Kenya OR Kyrgyzstan OR "Kyrgyz republic" OR Kyrgyzstan OR Kirghizia OR Kirghiz OR Kirgizstan OR Laos OR "Lao PDR" OR Lesotho OR Mauritania OR Mauritius OR Micronesia OR Mongolia OR Morocco OR Ifni OR Myanmar OR Burma OR Namibia OR Nicaragua OR Nigeria OR Pakistan OR "Papua New Guinea" OR Philippines OR Philipines OR Phillipines OR Phillippines OR Samoa OR "Sao Tome" OR Principe OR "Atlantic Islands" OR "Solomon Islands" OR Melanesia OR "Sri Lanka" OR Ceylon OR Swaziland OR Eswatini OR Syria OR "Syrian Arab Republic" OR "Tajikistan" OR Tadzhikistan OR Tadjikistan OR Tadzhik OR "East Timor" OR "Timor Leste" OR Tunisia OR Ukraine OR Uzbekistan OR Uzbek OR

		Vanuatu OR "New Hebrides" OR Vietnam OR "Viet Nam" OR "West Bank" OR Gaza OR Yemen OR Sanaa OR Zambia))
#4		#1 AND #2 AND #3
#5	Limit: Publication Year & Language	#4 AND (PY=(2011-2024)) AND (LA=("ENGLISH"))
#6	Limit: Publication Type	#5 NOT DT=(Review)

* Science Citation Index Expanded (SCI-EXPANDED)--1900-present
Social Sciences Citation Index (SSCI)--1900-present
Conference Proceedings Citation Index – Science (CPCI-S)--1990-present
Conference Proceedings Citation Index – Social Science & Humanities (CPCI-SSH)--1990-present
Book Citation Index – Science (BKCI-S)--2005-present
Book Citation Index – Social Sciences & Humanities (BKCI-SSH)--2005-present
Emerging Sources Citation Index (ESCI)--2005-present
Current Chemical Reactions (CCR-EXPANDED)--1985-present
Index Chemicus (IC)--1993-present

Database: Embase
Platform: Elsevier
Date Searched: August 3, 2021 / **Date Search Updated:** January 23, 2023 / **Date Search Updated:** August 5, 2024
Date Limits: Publication Year: 2011–2024
Other Limits/Filters: Language: English; Source: Embase

Set	Concept	Search Strategy
#1	Cancer	(neoplasm*:ti,ab OR cancer*:ti,ab OR oncolog*:ti,ab OR carcinoma*:ti,ab OR sarcoma*:ti,ab OR adenocarcinoma*:ti,ab OR malignan*:ti,ab OR neoplasia*:ti,ab OR leukemia*:ti,ab OR leukaemia*:ti,ab OR lymphoma*:ti,ab OR melanoma*:ti,ab OR myeloma*:ti,ab OR 'malignant neoplasm'/exp OR 'sarcoma'/exp OR 'adenosarcoma'/exp OR 'carcinoma'/de OR 'leukemia'/exp OR 'lymphoma'/exp OR 'melanoma'/exp)
#2	Survivorship	(survivorship:ti,ab OR aftercare:ti,ab OR 'after care':ti,ab OR 'followup care':ti,ab OR 'follow up care':ti,ab OR 'post treatment surveillance':ti,ab OR 'posttreatment surveillance':ti,ab OR 'post treatment care':ti,ab OR 'posttreatment care':ti,ab OR 'cancer survivor*':ti,ab OR 'cancer recurrence*':ti,ab OR 'longterm side effect*':ti,ab OR 'long term side effect*':ti,ab OR 'late term side effect*':ti,ab OR 'late side effect*':ti,ab OR 'primary health care':ti,ab OR 'continuity of care':ti,ab OR 'care continuity':ti,ab OR 'care continuum':ti,ab OR 'primary care':ti,ab OR 'patient care plan*':ti,ab OR 'case management':ti,ab OR 'care coordination':ti,ab OR 'coordinating care':ti,ab OR 'coordinated care':ti,ab OR 'financial toxicit*':ti,ab OR 'financially toxic*':ti,ab OR bankruptc*:ti,ab OR 'financial stress*':ti,ab OR 'financial distress':ti,ab OR 'workforce reentry':ti,ab OR 'workforce re entry':ti,ab OR 'return to work':ti,ab OR 'back to work':ti,ab OR 'quality of life':ti,ab OR 'family stress*':ti,ab OR 'family relationship*':ti,ab OR 'spousal relationship*':ti,ab OR 'spouse relation*':ti,ab OR 'lifestyle chang*':ti,ab OR 'life style chang*':ti,ab OR 'lifestyle modification*':ti,ab OR 'life style modification*':ti,ab OR 'behavior chang*':ti,ab OR 'behaviour chang*':ti,ab OR 'changing behavior*':ti,ab OR 'changing behaviour*':ti,ab OR stigma:ti,ab OR 'patient navigation':ti,ab OR 'patient navigator':ti,ab OR 'survivorship'/de OR 'survivor'/de OR 'cancer survivor'/de OR aftercare/de OR 'follow up'/mj OR 'cancer recurrence'/mj OR 'case management'/mj OR 'patient care planning'/mj OR 'financial distress'/mj OR 'financial stress'/mj OR 'return to work'/mj OR 'quality of life'/mj OR 'family relationships'/mj OR 'family stress'/mj OR 'behavior change'/mj OR 'lifestyle modification'/mj OR 'stigma'/mj OR 'patient navigator'/mj)
#3	Geographical Focus	('Developing Countries'/de OR 'low resource':ti,ab OR 'developing country':ti,ab OR 'developing countries':ti,ab OR LMIC:ti,ab OR LMICs:ti,ab OR 'lower middle income':ti,ab OR 'lmics':ti,ab OR 'lower middle income':ti,ab OR 'lower middle income countries':ti,ab OR 'lower middle income country':ti,ab OR 'lower middle income economies':ti,ab OR 'lower middle income setting':ti,ab OR

		'lower middle income settings':ti,ab OR 'low and middle income countries':ti,ab OR 'low and middle income country':ti,ab OR 'low and middle income':ti,ab OR 'low and middle income':ti,ab OR 'developing nation':ti,ab OR 'developing nations':ti,ab OR 'developing population':ti,ab OR 'developing populations':ti,ab OR 'developing world':ti,ab OR 'less developed country':ti,ab OR 'less developed countries':ti,ab OR 'less developed nation':ti,ab OR 'less developed nations':ti,ab OR 'less developed world':ti,ab OR 'lesser developed countries':ti,ab OR 'lesser developed nations':ti,ab OR 'under developed country':ti,ab OR 'under developed countries':ti,ab OR 'under developed nations':ti,ab OR 'under developed world':ti,ab OR 'underdeveloped country':ti,ab OR 'underdeveloped countries':ti,ab OR 'underdeveloped nation':ti,ab OR 'underdeveloped nations':ti,ab OR 'underdeveloped population':ti,ab OR 'underdeveloped populations':ti,ab OR 'underdeveloped world':ti,ab OR 'middle income country':ti,ab OR 'middle income countries':ti,ab OR 'middle income nation':ti,ab OR 'middle income nations':ti,ab OR 'middle income population':ti,ab OR 'middle income populations':ti,ab OR 'low income country':ti,ab OR 'low income countries':ti,ab OR 'low income nation':ti,ab OR 'low income nations':ti,ab OR 'low income population':ti,ab OR 'low income populations':ti,ab OR 'lower income country':ti,ab OR 'lower income countries':ti,ab OR 'lower income nations':ti,ab OR 'lower income population':ti,ab OR 'lower income populations':ti,ab OR 'underserved countries':ti,ab OR 'underserved nations':ti,ab OR 'deprived countries':ti,ab OR 'deprived population':ti,ab OR 'deprived populations':ti,ab OR 'poor country':ti,ab OR 'poor countries':ti,ab OR 'poor nation':ti,ab OR 'poor nations':ti,ab OR 'poor population':ti,ab OR 'poor populations':ti,ab OR 'poor world':ti,ab OR 'poorer countries':ti,ab OR 'poorer nations':ti,ab OR 'poorer population':ti,ab OR 'poorer populations':ti,ab OR 'developing economy':ti,ab OR 'developing economies':ti,ab OR 'less developed economy':ti,ab OR 'less developed economies':ti,ab OR 'underdeveloped economies':ti,ab OR 'middle income economy':ti,ab OR 'middle income economies':ti,ab OR 'low income economy':ti,ab OR 'low income economies':ti,ab OR 'lower income economies':ti,ab OR 'low gdp':ti,ab OR 'low gnp':ti,ab OR 'low gross domestic':ti,ab OR 'low gross national':ti,ab OR 'lower gdp':ti,ab OR 'lower gross domestic':ti,ab OR lmic:ti,ab OR lmics:ti,ab OR 'third world':ti,ab OR 'lami country':ti,ab OR 'lami countries':ti,ab OR 'transitional country':ti,ab OR 'transitional countries':ti,ab OR 'sub saharan africa':ti,ab OR 'africa south of the sahara'/de OR 'africa, central'/de OR 'central africa':ti,ab OR 'east africa':ti,ab OR 'eastern africa':ti,ab OR 'africa, eastern'/de OR 'southern africa':ti,ab OR 'africa, southern'/de OR 'western africa':ti,ab OR 'africa, western'/de OR 'west africa':ti,ab OR 'north africa':ti,ab OR 'northern africa':ti,ab OR 'africa, northern'/de OR 'asia, southeastern'/de OR 'asia, western'/de OR 'west

		asia':ti,ab OR 'south east asia':ti,ab OR 'southeast asia':ti,ab OR 'southeastern asia':ti,ab OR 'south america':ti,ab OR 'latin america':ti,ab OR 'central america':ti,ab OR africa:ti,ab OR asia:ti,ab OR caribbean:ti,ab OR 'west indies':ti,ab OR 'central america'/de OR 'latin america'/de OR 'south america'/de OR afghanistan:ti,ab OR 'afghanistan'/de OR algeria:ti,ab OR 'algeria'/de OR 'belize'/de OR belize:ti,ab OR benin:ti,ab OR 'benin'/de OR botswana:ti,ab OR 'botswana'/de OR 'burkina faso':ti,ab OR 'burkina fasso':ti,ab OR 'upper volta':ti,ab OR 'burkina faso'/de OR 'burundi'/de OR burundi:ti,ab OR urundi:ti,ab OR 'central african republic':ti,ab OR 'central african republic'/de OR 'chad'/de OR chad:ti,ab OR comoros:ti,ab OR 'comoro islands':ti,ab OR comores:ti,ab OR mayotte:ti,ab OR 'democratic republic of the congo'/de OR eritrea:ti,ab OR 'eritrea'/de OR eswatini:ti,ab OR 'eswatini'/de OR ethiopia:ti,ab OR 'ethiopia'/de OR gambia:ti,ab OR 'gambia'/de OR guinea:ti,ab OR 'guinea'/de OR 'guinea-bissau':ti,ab OR 'guinea-bissau'/de OR haiti:ti,ab OR 'haiti'/de OR 'north korea':ti,ab OR 'north korea'/de OR 'democratic people s republic of korea':ti,ab OR liberia:ti,ab OR 'liberia'/de OR Libya:ti,ab OR Libya/de OR madagascar:ti,ab OR 'madagascar'/de OR malawi:ti,ab OR 'malawi'/de OR nyasaland:ti,ab OR mali:ti,ab OR 'mali'/de OR mozambique:ti,ab OR 'mozambique'/de OR nepal:ti,ab OR 'nepal'/de OR niger:ti,ab OR 'niger'/de OR rwanda:ti,ab OR 'rwanda'/de OR senegal:ti,ab OR 'senegal'/de OR seychelles:ti,ab OR 'seychelles'/de OR 'sierra leone':ti,ab OR 'sierra leone'/de OR somalia:ti,ab OR 'somalia'/de OR 'south africa':ti,ab OR 'south africa'/de OR 'south sudan':ti,ab OR 'south sudan'/de OR 'sudan'/de OR sudan:ti,ab OR tanzania:ti,ab OR 'tanzania'/de OR 'togo'/de OR togo:ti,ab OR 'togolese republic':ti,ab OR uganda:ti,ab OR 'uganda'/de OR 'zimbabwe'/de OR zimbabwe:ti,ab OR rhodesia:ti,ab OR 'angola'/de OR angola:ti,ab OR 'bangladesh'/de OR bangladesh:ti,ab OR 'bhutan'/de OR bhutan:ti,ab OR 'bolivia'/de OR bolivia:ti,ab OR 'cabo verde':ti,ab OR 'cape verde':ti,ab OR 'cabo verde'/de OR 'cambodia'/de OR cambodia:ti,ab OR 'khmer republic':ti,ab OR kampuchea:ti,ab OR cameroon:ti,ab OR cameroons:ti,ab OR cameron:ti,ab OR 'cameroon'/de OR congo:ti,ab OR zaire:ti,ab OR 'congo'/de OR 'cote d ivoire':ti,ab OR 'ivory coast':ti,ab OR 'cote divoire':ti,ab OR 'djibouti'/de OR djibouti:ti,ab OR 'french somaliland':ti,ab OR egypt:ti,ab OR 'united arab republic':ti,ab OR 'egypt'/de OR 'el salvador':ti,ab OR 'el salvador'/de OR 'equatorial guinea':ti,ab OR 'equatorial guinea'/de OR gabon:ti,ab OR 'gabon'/de OR ghana:ti,ab OR 'ghana'/de OR 'gold coast':ti,ab OR honduras:ti,ab OR 'honduras'/de OR 'india'/de OR india:ti,ab OR kashmir:ti,ab OR indonesia:ti,ab OR 'indonesia'/de OR iran:ti,ab OR 'iran'/de OR kenya:ti,ab OR 'kenya'/de OR kiribati:ti,ab OR 'kyrgyzstan'/de OR 'kyrgyz republic':ti,ab OR kyrgyzstan:ti,ab OR kirghizia:ti,ab OR kirghiz:ti,ab OR kirgizstan:ti,ab OR laos:ti,ab OR 'lao pdr':ti,ab OR 'laos'/de OR lesotho:ti,ab OR 'lesotho'/de OR mauritania:ti,ab OR

		'mauritania'/de OR mauritius:ti,ab OR 'mauritius'/de OR micronesia:ti,ab OR 'micronesia'/de OR mongolia:ti,ab OR 'mongolia'/de OR morocco:ti,ab OR ifni:ti,ab OR 'morocco'/de OR myanmar:ti,ab OR burma:ti,ab OR 'myanmar'/de OR namibia:ti,ab OR 'namibia'/de OR nicaragua:ti,ab OR 'nicaragua'/de OR 'nigeria'/de OR nigeria:ti,ab OR pakistan:ti,ab OR 'pakistan'/de OR 'papua new guinea':ti,ab OR 'papua new guinea'/de OR philippines:ti,ab OR philipines:ti,ab OR phillipines:ti,ab OR phillippines:ti,ab OR 'philippines'/de OR samoa:ti,ab OR 'samoa'/de OR 'sao tome':ti,ab OR principe:ti,ab OR 'sao tome and principe'/de OR 'solomon islands':ti,ab OR melanesia:ti,ab OR 'sri lanka':ti,ab OR ceylon:ti,ab OR 'sri lanka'/de OR swaziland:ti,ab OR 'swaziland'/de OR 'eswatini'/de OR eswatini:ti,ab OR syria:ti,ab OR 'syria'/de OR 'syrian arab republic':ti,ab OR tajikistan:ti,ab OR 'tajikistan'/de OR tadzhikistan:ti,ab OR tadjikistan:ti,ab OR tadzhik:ti,ab OR 'east timor':ti,ab OR 'timor leste':ti,ab OR 'timor-leste'/de OR tunisia:ti,ab OR 'tunisia'/de OR ukraine:ti,ab OR 'ukraine'/de OR uzbekistan:ti,ab OR 'uzbekistan'/de OR uzbek:ti,ab OR vanuatu:ti,ab OR 'vanuatu'/de OR 'new hebrides':ti,ab OR vietnam:ti,ab OR 'viet nam':ti,ab OR 'vietnam'/de OR 'west bank':ti,ab OR 'gaza strip':ti,ab OR gaza:ti,ab OR yemen:ti,ab OR 'yemen'/de OR sanaa:ti,ab OR zambia:ti,ab OR 'zambia'/de)
#4		#1 AND #2 AND #3
#5	Limit: Publication Year & Language	#4 AND ([embase]/lim AND [english]/lim AND [2021-2024]/py)
#6	Limit: Publication Type	#5 NOT ([systematic review]/lim OR [meta analysis]/lim OR 'review'/exp OR 'systematic review'/exp OR review:ti,ab OR metanalysis:ti OR 'meta analysis':ti OR 'meta analyses':ti OR [review]/lim)
#7	Limit: Exclude MEDLINE	#6 NOT [medline]/lim

Notes: The limits for language (English) and publication year (2011–2024) were applied to the main search using the filters available. The keywords were searched in the title and abstract fields (i.e., :ti,ab), title field (i.e., :ti), and the controlled vocabulary terms are indicated with (i.e., /de) as Descriptor, (i.e., /exp) as Explode an EMTREE term, or as a Major EMTREE term (i.e., /mj). Phrases were enclosed in quotation marks to force the searching of the exact terms in order presented. To these results, the search strategy to exclude publication types not of interest as per eligibility criteria was used. No other limits were applied to the searches.

Database: CINAHL Plus
Platform: EBSCOhost
Date Searched: August 3, 2021 / **Date Search Updated:** January 23, 2023 / **Date Search Updated:** August 5, 2024
Date Limits: Publication Year: 2011–2024
Other Limits/Filters: Language: English

Set	Concept	Search Strategy
#1	Cancer	Title: (neoplasm* OR cancer* OR oncolog* OR carcinoma* OR sarcoma* OR adenocarcinoma* OR malignan* OR neoplasia* OR leukemia* OR leukaemia* OR lymphoma* OR melanoma* OR myeloma*)
#2	Cancer	Abstract: (neoplasm* OR cancer* OR oncolog* OR carcinoma* OR sarcoma* OR adenocarcinoma* OR malignan* OR neoplasia* OR leukemia* OR leukaemia* OR lymphoma* OR melanoma* OR myeloma*)
#3	Cancer	Exact Subject Heading: (MH "Neoplasms") OR (MH "Leukemia+") OR (MH "Lymphoma+") OR (MH "Melanoma+") OR (MH "Sarcoma") OR (MH "Adenocarcinoma") OR (MH "Carcinoma")
#4	Cancer	#1 OR #2 OR #3
#5	Survivorship	Title: (survivorship OR aftercare OR “after care” OR “followup care” OR “follow up care” OR “post treatment surveillance” OR “posttreatment surveillance” OR “post treatment care” OR “posttreatment care” OR “cancer survivor” OR “cancer survivors” OR “cancer recurrence” OR “cancer recurrences” OR “longterm side effect” OR “longterm side effects” OR “long term side effect” OR “long term side effects” OR “late term side effect” OR “late term side effects” OR “late side effect” OR “late side effects” OR “primary health care” OR “continuity of care” OR “care continuity” OR “care continuum” OR “primary care” OR “patient care plan” OR “patient care plans” OR “patient care planning” OR “case management” OR “care coordination” OR “coordinating care” OR “coordinated care” OR “financial toxicity” OR “financially toxic” OR bankruptcy OR “financial stress” OR “financial stresses” OR “financial distress” OR “workforce reentry” OR “workforce re entry” OR “return to work” OR “back to work” OR “quality of life” OR “family stress” OR “family stresses” OR “family relationship” OR “family relationships” OR “spousal relationship” OR “spousal relationships” OR “spouse relations” OR “lifestyle change” OR “lifestyle changes” OR “life style change” OR “life style changes” OR “lifestyle modification” OR “lifestyle modifications” OR “life style modification” OR “life style modifications” OR “behavior change” OR “behavior changes” OR “behaviour change” OR “behaviour changes” OR “changing behavior” OR “changing behaviors” OR stigma OR “patient navigation” OR “patient navigator”)

#6	Survivorship	Abstract: (survivorship OR aftercare OR “after care” OR “followup care” OR “follow up care” OR “post treatment surveillance” OR “posttreatment surveillance” OR “post treatment care” OR “posttreatment care” OR “cancer survivor” OR “cancer survivors” OR “cancer recurrence” OR “cancer recurrences” OR “longterm side effect” OR “longterm side effects” OR “long term side effect” OR “long term side effects” OR “late term side effect” OR “late term side effects” OR “late side effect” OR “late side effects” OR “primary health care” OR “continuity of care” OR “care continuity” OR “care continuum” OR “primary care” OR “patient care plan” OR “patient care plans” OR “patient care planning” OR “case management” OR “care coordination” OR “coordinating care” OR “coordinated care” OR “financial toxicity” OR “financially toxic” OR bankruptcy OR “financial stress” OR “financial stresses” OR “financial distress” OR “workforce reentry” OR “workforce re entry” OR “return to work” OR “back to work” OR “quality of life” OR “family stress” OR “family stresses” OR “family relationship” OR “family relationships” OR “spousal relationship” OR “spousal relationships” OR “spouse relations” OR “lifestyle change” OR “lifestyle changes” OR “life style change” OR “life style changes” OR “lifestyle modification” OR “lifestyle modifications” OR “life style modification” OR “life style modifications” OR “behavior change” OR “behavior changes” OR “behaviour change” OR “behaviour changes” OR “changing behavior” OR “changing behaviors” OR stigma OR “patient navigation” OR “patient navigator”)
#7	Survivorship	Exact Subject Headings: (MH "Survivorship") OR (MH "Cancer Survivors") OR (MH "Survivors") OR (MH "After Care") OR (MM "Case Management") OR (MM "Patient Care Plans") OR (MM "Financial Stress") OR (MM "Job Re-Entry") OR (MM "Quality of Life") OR (MM "Family Relations") OR (MM "Behavioral Changes") OR (MM "Life Style Changes") OR (MM "Stigma") OR (MM "Patient Navigation")
#8	Survivorship	#5 OR #6 OR #7
#9	Geographical Focus	Title: (“low resource” OR “developing country” OR “developing countries” OR LMIC OR LMICs OR "lower middle income" OR "lower middle income" OR "lower middle income countries" OR "lower middle income country" OR "lower middle income economies" OR "lower middle income setting" OR "lower middle income settings" OR "developing nation" OR "developing nations" OR "developing population" OR "developing populations" OR "developing world" OR "less developed country" OR "less developed countries" OR "less developed nation" OR "less developed nations" OR "less developed world" OR "lesser developed countries" OR "lesser developed nations" OR "under developed country" OR "under developed countries" OR "under developed nations" OR "under developed world" OR "underdeveloped country" OR "underdeveloped countries" OR "underdeveloped nation" OR "underdeveloped nations" OR "underdeveloped population" OR "underdeveloped populations"

		OR "underdeveloped world" OR "middle income country" OR "middle income countries" OR "middle income nation" OR "middle income nations" OR "middle income population" OR "middle income populations" OR "low income country" OR "low income countries" OR "low income nation" OR "low income nations" OR "low income population" OR "low income populations" OR "lower income country" OR "lower income countries" OR "lower income nations" OR "lower income population" OR "lower income populations" OR "underserved countries" OR "underserved nations" OR "poor country" OR "poor countries" OR "poor nation" OR "poor nations" OR "poor population" OR "poor populations" OR "poor world" OR "poorer countries" OR "poorer nations" OR "poorer population" OR "poorer populations" OR "developing economy" OR "developing economies" OR "less developed economy" OR "less developed economies" OR "underdeveloped economies" OR "middle income economy" OR "middle income economies" OR "low income economy" OR "low income economies" OR "lower income economies" OR "low gdp" OR "low gnp" OR "low gross domestic" OR "low gross national" OR "lower gdp" OR "lower gross domestic" OR lmic OR lmics OR "third world" OR "lami country" OR "lami countries" OR "transitional country" OR "transitional countries" OR "sub Saharan Africa" OR "sub-saharan Africa" OR "Africa South of the Sahara" OR “central Africa” OR “East Africa” OR “Eastern Africa” OR “Southern Africa” OR “Western Africa” OR “West Africa” OR “north Africa” OR “Northern Africa” OR “West Asia” OR “South East Asia” OR “southeast asia” OR “southeastern asia” OR "South America" OR "Latin America" OR "Central America" OR “West Indies” OR Afghanistan OR Algeria OR Belize OR Benin OR Botswana OR “Burkina Fasso” OR “Upper Volta” OR “Burkina Faso” OR Burundi OR Urundi OR “Central African Republic” OR Chad OR Comoros OR “Comoro Islands” OR Comores OR Mayotte OR “Democratic Republic of the Congo” OR Eritrea OR Eswatini OR Ethiopia OR Gambia OR Guinea OR “Guinea-Bissau” OR Haiti OR “North Korea” OR "Democratic People's Republic of Korea" OR Liberia OR Madagascar OR Malawi OR Nyasaland OR Mali OR Mozambique OR Nepal OR Niger OR Rwanda OR Senegal OR Seychelles OR “Sierra Leone” OR Somalia OR “South Africa” OR “South Sudan” OR Sudan OR Tanzania OR Togo OR “Togolese Republic” OR Uganda OR Zimbabwe OR Rhodesia OR Angola OR Bangladesh OR Bhutan OR Bolivia OR “cabo verde” OR “cape verde” OR Cambodia OR “Khmer Republic” OR Kampuchea OR Cameroon OR Cameroons OR Cameron OR Zaire OR Congo OR “Cote d'Ivoire” OR “Ivory Coast” OR Djibouti OR “French Somaliland” OR Egypt OR “United Arab Republic” OR “el salvador” OR “Equatorial Guinea” OR Gabon OR Ghana OR “Gold Coast” OR Honduras OR India OR Kashmir OR Indonesia OR Iran OR Kenya OR Kyrgyzstan OR “Kyrgyz republic” OR Kyrgyzstan OR Kirghizia OR Kirghiz OR Kirgizstan OR Laos OR "Lao PDR" OR Lesotho OR Libya OR Mauritania OR Mauritius OR Micronesia OR Mongolia OR Morocco OR Ifni OR Myanmar OR Burma OR Namibia OR Nicaragua OR Nigeria OR Pakistan OR “Papua New Guinea” OR Philippines OR Philipines OR Phillipines OR

		Phillippines OR Samoa OR “Sao Tome” OR Principe OR "Atlantic Islands" OR “Solomon Islands” OR Melanesia OR “Sri Lanka” OR Ceylon OR Swaziland OR Eswatini OR Syria OR “Syrian Arab Republic” OR "Tajikistan" OR Tadzhikistan OR Tadjikistan OR Tadzhik OR “East Timor” OR “Timor Leste” OR Tunisia OR Ukraine OR Uzbekistan OR Uzbek OR Vanuatu OR “New Hebrides” OR Vietnam OR “Viet Nam” OR “West Bank” OR Gaza OR Yemen OR Sanaa OR Zambia)
#10	Geographical Focus	Abstract: (“low resource” OR “developing country” OR “developing countries” OR LMIC OR LMICs OR "lower middle income" OR "lower middle income" OR "lower middle income countries" OR "lower middle income country" OR "lower middle income economies" OR "lower middle income setting" OR "lower middle income settings" OR "developing nation" OR "developing nations" OR "developing population" OR "developing populations" OR "developing world" OR "less developed country" OR "less developed countries" OR "less developed nation" OR "less developed nations" OR "less developed world" OR "lesser developed countries" OR "lesser developed nations" OR "under developed country" OR "under developed countries" OR "under developed nations" OR "under developed world" OR "underdeveloped country" OR "underdeveloped countries" OR "underdeveloped nation" OR "underdeveloped nations" OR "underdeveloped population" OR "underdeveloped populations" OR "underdeveloped world" OR "middle income country" OR "middle income countries" OR "middle income nation" OR "middle income nations" OR "middle income population" OR "middle income populations" OR "low income country" OR "low income countries" OR "low income nation" OR "low income nations" OR "low income population" OR "low income populations" OR "lower income country" OR "lower income countries" OR "lower income nations" OR "lower income population" OR "lower income populations" OR "underserved countries" OR "underserved nations" OR "poor country" OR "poor countries" OR "poor nation" OR "poor nations" OR "poor population" OR "poor populations" OR "poor world" OR "poorer countries" OR "poorer nations" OR "poorer population" OR "poorer populations" OR "developing economy" OR "developing economies" OR "less developed economy" OR "less developed economies" OR "underdeveloped economies" OR "middle income economy" OR "middle income economies" OR "low income economy" OR "low income economies" OR "lower income economies" OR "low gdp" OR "low gnp" OR "low gross domestic" OR "low gross national" OR "lower gdp" OR "lower gross domestic" OR lmic OR lmics OR "third world" OR "lami country" OR "lami countries" OR "transitional country" OR "transitional countries" OR "sub Saharan Africa" OR "sub-saharan Africa" OR "Africa South of the Sahara" OR “central Africa” OR “East Africa” OR “Eastern Africa” OR “Southern Africa” OR “Western Africa” OR “West Africa” OR “north Africa” OR “Northern Africa” OR “West Asia” OR “South East Asia” OR “southeast asia” OR “southeastern asia” OR "South America" OR "Latin America" OR "Central America" OR “West Indies” OR Afghanistan OR Algeria OR Belize OR Benin OR Botswana OR

		“Burkina Fasso” OR “Upper Volta” OR “Burkina Faso” OR Burundi OR Urundi OR “Central African Republic” OR Chad OR Comoros OR “Comoro Islands” OR Comores OR Mayotte OR “Democratic Republic of the Congo” OR Eritrea OR Eswatini OR Ethiopia OR Gambia OR Guinea OR “Guinea-Bissau” OR Haiti OR “North Korea” OR "Democratic People's Republic of Korea" OR Liberia OR Libya OR Madagascar OR Malawi OR Nyasaland OR Mali OR Mozambique OR Nepal OR Niger OR Rwanda OR Senegal OR Seychelles OR “Sierra Leone” OR Somalia OR “South Africa” OR “South Sudan” OR Sudan OR Tanzania OR Togo OR “Togolese Republic” OR Uganda OR Zimbabwe OR Rhodesia OR Angola OR Bangladesh OR Bhutan OR Bolivia OR “cabo verde” OR “cape verde” OR Cambodia OR “Khmer Republic” OR Kampuchea OR Cameroon OR Cameroons OR Cameron OR Zaire OR Congo OR “Cote d'Ivoire” OR “Ivory Coast” OR Djibouti OR “French Somaliland” OR Egypt OR “United Arab Republic” OR “el salvador” OR “Equatorial Guinea” OR Gabon OR Ghana OR “Gold Coast” OR Honduras OR India OR Kashmir OR Indonesia OR Iran OR Kenya OR Kyrgyzstan OR “Kyrgyz republic” OR Kyrgyzstan OR Kirghizia OR Kirghiz OR Kirgizstan OR Laos OR "Lao PDR" OR Lesotho OR Mauritania OR Mauritius OR Micronesia OR Mongolia OR Morocco OR Ifni OR Myanmar OR Burma OR Namibia OR Nicaragua OR Nigeria OR Pakistan OR “Papua New Guinea” OR Philippines OR Philipines OR Phillipines OR Phillippines OR Samoa OR “Sao Tome” OR Principe OR "Atlantic Islands" OR “Solomon Islands” OR Melanesia OR “Sri Lanka” OR Ceylon OR Swaziland OR Eswatini OR Syria OR “Syrian Arab Republic” OR "Tajikistan" OR Tadzhikistan OR Tadjikistan OR Tadzhik OR “East Timor” OR “Timor Leste” OR Tunisia OR Ukraine OR Uzbekistan OR Uzbek OR Vanuatu OR “New Hebrides” OR Vietnam OR “Viet Nam” OR “West Bank” OR Gaza OR Yemen OR Sanaa OR Zambia)
#11	Geographical Focus	Exact Subject Heading: (MH "Developing Countries") OR (MH "Africa South of the Sahara+") OR (MH "Africa, Northern+") OR (MH "Algeria") OR (MH "Egypt") OR (MH "Libya") OR (MH "Morocco") OR (MH "Tunisia") OR (MH "Africa, Central+") OR (MH "Africa, Eastern+") OR (MH "Africa, Southern+") OR (MH "Africa, Western+") OR (MH "Africa+") OR (MH "Asia, Central") OR (MH "Kyrgyzstan") OR (MH "Tajikistan") OR (MH "Turkmenistan") OR (MH "Uzbekistan") OR (MH "Benin") OR (MH "Burkina Faso") OR (MH "Cape Verde") OR (MH "Cote d'Ivoire") OR (MH "Gambia") OR (MH "Ghana") OR (MH "Guinea") OR (MH "Guinea-Bissau") OR (MH "Liberia") OR (MH "Mali") OR (MH "Mauritania") OR (MH "Niger") OR (MH "Nigeria") OR (MH "Senegal") OR (MH "Sierra Leone") OR (MH "Togo") OR (MH "Zimbabwe") OR (MH "Zambia") OR (MH "South Africa") OR (MH "Swaziland") OR (MH "Namibia") OR (MH "Mozambique") OR (MH "Malawi") OR (MH "Lesotho") OR (MH "Botswana") OR (MH "Angola") OR (MH "Uganda") OR (MH "Tanzania") OR (MH "Sudan") OR (MH "Somalia") OR (MH "Rwanda") OR (MH "Ethiopia") OR (MH "Kenya") OR (MH "Eritrea") OR (MH "Djibouti") OR (MH "Burundi") OR (MH "Cameroon") OR (MH "Central African Republic") OR (MH "Chad") OR (MH "Congo") OR (MH

		"Democratic Republic of the Congo") OR (MH "Gabon") OR (MH "Equatorial Guinea") OR (MH "Cambodia") OR (MH "East Timor") OR (MH "Indonesia") OR (MH "Laos") OR (MH "Myanmar") OR (MH "Philippines") OR (MH "Vietnam") OR (MH "Asia, Southeastern") OR (MH "Bangladesh") OR (MH "Bhutan") OR (MH "India") OR (MH "Nepal") OR (MH "Pakistan") OR (MH "Sri Lanka") OR (MH "Middle East") OR (MH "Afghanistan") OR (MH "Iran") OR (MH "Iraq") OR (MH "Syria") OR (MH "Yemen") OR (MH "North Korea") OR (MH "Libya") OR (MH "Mongolia") OR (MH "Ukraine") OR (MH "Madagascar") OR (MH "Low and Middle Income Countries") OR (MM "Melanesia") OR (MH "Papua New Guinea") OR (MH "Samoa") OR (MH "Independent State of Samoa")
#12	Geographical Focus	#9 OR #10 OR #11
#13	Limit: Publication Type	Title: ("systematic review*" OR "meta analysis" OR "meta analyses" OR metanalyses OR metanalysis)
#14	Limit: Publication Type	Abstract: ("systematic review*" OR "meta analysis" OR "meta analyses" OR metanalyses OR metanalysis)
#15	Limit: Publication Type	Exact Subject Heading: (MH "Scoping Review" OR MH "Literature Review" OR MH "Meta Analysis" OR MH "Systematic Review")
#16	Limit: Publication Type	#13 OR #14 OR #15
#17		#4 AND #8 AND #12
#18	Limit: Publication Year & Language	#17 AND Limits: Publication Year & Language: English
#19	Limit: Publication Type	#18 NOT #16

Notes: The limits for language (English) and publication year (2011–2024) were applied to the main search using the filters available. The keywords were searched in the title and abstract fields and the controlled vocabulary terms are indicated with MH and searched in the Exact Subject Heading. Phrases were enclosed in quotation marks to force the searching of the exact terms in order

presented. To these results, the search strategy to exclude publication types not of interest as per eligibility criteria was used. No other limits were applied to the searches.
