# Supplementary material for: A scoping review exploring cancer survivorship in Africa: 2011 to 2024
Source: J Cancer Surviv. 2025 Apr 25;20(Suppl 1):1127–37. doi: 10.1007/s11764-025-01805-5 (PMC13676652; doi:10.1007/s11764-025-01805-5)
Supplement: Supplementary file 2 [file 11764_2025_1805_MOESM2_ESM.pdf]

Basic Study Information

Article Title

Title of paper / abstract / report that data are extracted from

[ ]

Publication year

Please write as XXXX. Should be published within the range of 2011 to 2022.

[ ]

Is this a conference abstract or full journal article?

- ○ Conference abstract
- ○ Journal article
- ○ Other/unsure

Before continuing with data extraction, please double-check whether the article should be excluded by referring to the eligibility criteria .

Eligibility criteria: https://docs.google.com/document/d/1TIeNowC7g9EWSViOE-jCnlACbbENkI3y7b_tOaDC7go/edit?usp=sharing

If it should be excluded, please indicate that here and why.

If it should be included, please continue with the data extraction for this article.

[ ]

Study type/design (select all that apply)

- ☐ Qualitative (focus group discussion, key informant interviews or ethnography)
- ☐ Quantitative (survey, cohort study, quasi-experimental, pre-post intervention, etc.)
- ☐ Mixed-methods (combination of qualitative and quantitative)
- ☐ Program description
- ☐ Unsure
- ☐ Other

[ ]

Study setting

- ○ Cancer center
- ○ General hospital

- Health clinic
- Community based
- Home-based
- Other

Characteristics of Included Studies

African country in which the study was conducted

We are limiting to the African continent.

Please write out the full name of the country the study was conducted in. If multiple countries are reported from Africa, please add a semicolon (;) between each country name.

Study population

If more than one group makes up the study population, select all that apply.

- Survivors
- Caregivers (family, friends, members of community who are providing care to the patient)
- Oncology specialists (physicians)
- Oncology specialists (nurses, pharmacists, social workers, etc.)
- Healthcare workers not specialized in oncology
- Community health workers
- Policymakers
- Advocates
- Patient navigators
- Community leaders
- Other

Type of cancer studied (select all that apply)

If a general umbrella term is used (e.g., gynecological), look to the Results section to determine specific cancer types studied. If more specific terms not used, select Unspecified and add the general umbrella term to the Other field.

- Anal
- Bladder
- Brain
- Breast
- Cervix
- Endometrial

- [ ] Esophageal
- [ ] Ewing's
- [ ] Gastric (stomach)
- [ ] Gastroesophageal junction (GEJ)
- [ ] Germ cell
- [ ] Gestational trophoblastic neoplasm (GTN or GTD)
- [ ] Hepatocellular (liver)
- [ ] Head and neck
- [ ] Kaposi sarcoma
- [ ] Leukemia
- [ ] Lung
- [ ] Lymphoma
- [ ] Melanoma
- [ ] Mesothelioma
- [ ] Multiple myeloma
- [ ] Neuroendocrine
- [ ] Rhabdomyosarcoma
- [ ] Testicular
- [ ] Thyroid
- [ ] Uterine
- [ ] Vulvar
- [ ] Wilms Tumor (Nephroblastoma)
- [ ] Unspecified
- [ ] Other

Does this study primarily focus on survivors of pediatric cancers (age <15 at cancer diagnosis)?

- ○ Yes
- ○ No
- ○ Unsure

Survivorship Components

Based on the Institute of Medicine framework, which can be found here: https://www.nap.edu/catalog/11468/from-cancer-patient-to-cancer-survivor-lost-in-transition

Does the word "survivor" or "survivorship" appear in the study?

You can do a CTRL+F to search the article for survivor/survivors/survivorship. Should appear in the title, abstract, introduction, methods, results, discussion, or conclusion sections.

- ○ Yes
- ○ No

If survivors/survivorship is mentioned, are they defined?

Select N/A if survivorship or survivors are not mentioned in the study.

- Yes
- No
- N/A

If yes, what is the definition of survivorship or survivor used in the study?

Copy and paste verbatim with quotation marks and page number from the text if possible, otherwise denote as a summary and briefly summarize. Include origin citation of definition if article is citing a defintion.

Does the article talk about *POST-TREATMENT SURVEILLANCE* for recurrent or subsequent primary cancer?

- Yes
- No
- Unsure

Does the article talk about *PHYSICAL* long- or late-term effects of cancer and its treatment?

- Yes
- No
- Unsure

If yes, which *PHYSICAL* effects are discussed?

Select all that are discussed in the article.

- [ ] Anorexia/loss of appetite
- [ ] Assistive equipment (e.g., ostomy bag, crutches, prosthesis, etc.)
- [ ] Cardiotoxicity (heart issues, palpitations, dizziness)
- [ ] Constipation
- [ ] Infertility
- [ ] Lung toxicity (breathlessness, difficulty breathing, cough)
- [ ] Lymphedema (arm swelling)
- [ ] Malnutrition/cachexia
- [ ] Mobility/musculoskeletal issues
- [ ] Nausea/vomiting/diarrhea
- [ ] Neurological/cognitive deficits (IQ, forgetfulness, brain fog, chemo brain, etc.)
- [ ] Neuropathy
- [ ] Obesity
- [ ] Pain
- [ ] Sensory changes (tinnitus, ringing in the ears, changes to taste, changes in ability to smell, vision changes)

- [ ] Sexual health
- [ ] Sleeping difficulty
- [ ] Weakness
- [ ] Other

Does the article talk about *PSYCHOSOCIAL* long- or late-term effects of cancer and its treatment?

- ◯ Yes
- ◯ No
- ◯ Unsure

If yes, which *PSYCHOSOCIAL* effects are discussed?

Select all that are discussed in the article.

- [ ] Anxiety
- [ ] Body image alterations
- [ ] Community rejection
- [ ] Depression
- [ ] Fear of death
- [ ] Medical debt/financial toxicity
- [ ] Return to work difficulty
- [ ] Spousal abandonment
- [ ] Stigma
- [ ] Other

Does the article talk about *CARE COORDINATION* between different stakeholders (including specialist and non-specialist care providers)?

- ◯ Yes
- ◯ No
- ◯ Unsure

If yes, what is the model of *CARE COORDINATION* discussed in the article?

- [ ] Between oncology specialist and primary care providers
- [ ] Between oncology specialist and palliative care provider
- [ ] Between oncology specialist and other practitioners (nurse, patient navigator, CHW)
- [ ] Between oncology specialist and community leaders (complementary and alternative medicine practitioners, religious leaders)
- [ ] N/A
- [ ] Other

What key groups are mentioned in relation to survivorship care delivery or interventions?

These should only be included if they are a main focus of the article, not if they are simply mentioned.

- ☐ Cancer centers
- ☐ Caregivers (family, friends, community members who care for the survivors)
- ☐ Community health workers (CHW)
- ☐ Complementary and alternative medicine practitioners
- ☐ Faith-based organizations (FBOs)
- ☐ Home-based care providers
- ☐ Non-governmental organizations (NGOs)
- ☐ Nurses
- ☐ Patient navigators
- ☐ Peer survivors (survivor-led support groups, peer-to-peer support, survivor-led advocacy campaigns)
- ☐ Policymakers
- ☐ Primary care or general practitioners
- ☐ None
- ☐ Other

Is there an intervention in this study?

- ○ Yes
- ○ No
- ○ Unsure

If yes, summarize the intervention in one sentence maximum. Provide page numbers, if applicable.

Was quality of life (QOL) measured in the study via a questionnaire or survey?

- ○ Yes
- ○ No
- ○ Unsure

If yes, was measuring quality of life the primary purpose of the study?

- ○ Yes
- ○ No
- ○ Unsure

Final thoughts/comments on this article

Other comments

Please provide any final comments or thoughts on the article in relation to the purpose of this scoping review.
