# Supplementary material for: A scoping review exploring cancer survivorship in Africa: 2011 to 2024
Source: J Cancer Surviv. 2025 Apr 25;20(Suppl 1):1127–37. doi: 10.1007/s11764-025-01805-5 (PMC13676652; doi:10.1007/s11764-025-01805-5)
Supplement: Supplementary file 3 [file 11764_2025_1805_MOESM3_ESM.pdf]

Covidence #	Study ID	Title	Reviewer Name	Article Title	Publication year	Is this a conference abstract or full journal article?
Identification number given by Covidence for the study<br><br>Will not be necessary for analysis but to track specific data to its oriiginal study	First Author then year of publication	Original title of study as imported into Covidence	All fields here should be "Consensus"<br><br>If there was a reviewer name here that means we've chosen wrong fields	Title of study as entered by reviewer(s) and then chosen by consensus<br><br>This should be identical to cell in same row and column C	Year of publication, NOT submission or when study was accepted<br><br>For conference abstracts, this is the year of conference<br><br>This includes all years between and including 2011 and 2023	Only 2 options:<br>- "Journal Article"<br>- "Conference Abstract"

Before continuing with data extraction, please double-check whether the article should be excluded by referring to the eligibility criteria	Study type/design (select all that apply)	Study setting	African country in which the study was conducted
This column should be empty<br><br>- No study should have indication for exclusion here<br><br>- If a study had a note in this column to exclude then:<br>--Double check whether we discussed that study specifically and had a final group agreement to include, and delete the comment for exclusion here so it is not confusing<br>OR<br>--Study needs to be sent back into "Full Text Review" in Covidence and excluded there<br><br>- If in doubt, please mark study for group discussion and send email with identifiers of study (Columns A and B)<br><br>- Also, since some reviewers indicated the decision to exclude in other cells, please check every row using Ctrl+F and type "Exclu" and "Ineligible" to capture any variations. Same rules above apply	Only 4 options:<br>- "Quantitative"<br>- "Qualitative"<br>- "Mixed"<br>- "Program Description"<br><br>If more than one included, replace with "Mixed"<br><br>If "Unsure" or "Other" or "Unknown", please review study and fill in appropriately<br><br>If still unclear then put "Not reported"	This is the location/institute where participants were recruited, or if that is unclear, the institution with which the authors were affiliated<br><br>Only options:<br>- "Hospital, General"<br>- "Hospital, Academic"<br>- "Hospital, Other" (If not sure type of hospital)<br>- "University" (usually this is the option to choose if recruitment site is unclear and the author is affiliated with a university)<br>- "Cancer Center"<br>- "Community Based" (This includes NGOs, associations, and groups)<br>- "Social Media"<br>- "Health Clinic"<br>- "Home Based"<br>- "Other" (This is anything that does not fit in any of the above options)	Country in which study was conducted<br><br>If more than one country, then please separate them only with a comma without any other spaces<br><br>Example: "Egypt,Sudan,Zambia"

Study population	Type of cancer studied (select all that apply)	Does this study primarily focus on survivors of pediatric cancers (age <15 at cancer diagnosis)?
Only options:<br>- "Survivors"<br>- "Caregivers"<br>- "Oncology Specialists"<br>- "Healthcare Workers not Specialized in Oncology"<br>- "Community Health Workers"<br>- "Policymakers"<br>- "Advocates"<br>- "Patient Navigators"<br>- "Community Leaders"<br>- "Other" (Delete anything that comes afterward unless it fits in one of the previous other categories)<br><br>If more than one group included, please include all, and separate only by a comma without any spaces<br><br>Example: "Survivors,Oncology specialist,Pharmacists"	Indicate cancer type<br><br>This should be one of the following:<br>- "Leukemia" (No need to specify leukemia subtype like AML or CLL)<br>- "Lymphoma" (No need to specify lymphoma type like HL or Burkitt)<br>- "Multiple Myeloma"<br>- "Neuroblastoma"<br>- "Germ Cell Tumor" (If organ involved not specified)<br>- "Sarcoma" (Any tumor with sarcoma in the name)<br>- "Neuroendocrine Tumor"<br>- Name of an organ<br>- "Childhood Cancer" (If doesn't specify any further)<br>- "Gynecologic Cancer" (If doesn't specify any further)<br>- "Other" (If specified and doesn't meet any of the above, but no need to specify any further here)<br>- "Unspecified" (If it says cancer in general)<br><br>If Wilm's Tumor then put "Renal"<br>If Retinoblastoma then put "Eye"<br>If basal cell carcinoma, squamous cell carcinoma, or melanoma then put "Skin"<br><br>If more than one cancer included, please include all, and separate only by a comma without any spaces<br><br>Example: "Multiple Myeloma,Neuroblastoma,Leukemia"	Either "Yes" or "No"<br><br>If reviewer indicated "Other" or "Unsure", please check in original study, and enter either "Yes" or "No" in this column

Does the word "survivor" or "survivorship" appear in the study?	If survivors/survivorship is mentioned, are they defined?	If yes, what is the definition of survivorship or survivor used in the study?	Does the article talk about *POST-TREATMENT SURVEILLANCE* for recurrent or subsequent primary cancer?	Does the article talk about *PHYSICAL* long- or late-term effects of cancer and its treatment?
Either "Yes" or "No"<br><br>If reviewer indicated "Other" or "Unsure", please check in original study, and enter either "Yes" or "No" in this column	Either "Yes", "No", or "N/A"<br><br>If reviewer indicated "Other" or "Unsure", please check in original study, and enter either "Yes", "No", or "N/A" in this column	Definition should be entered here only if previous column answer is "Yes"<br><br>Otherwise, the value here should be "N/A"	Either "Yes" or "No"<br><br>If reviewer indicated "Other" or "Unsure", please check in original study, and enter either "Yes" or "No" in this column	Either "Yes" or "No"<br><br>If reviewer indicated "Other" or "Unsure", please check in original study, and enter either "Yes" or "No" in this column

If yes, which *PHYSICAL* effects are discussed?	Does the article talk about *PSYCHOSOCIAL* long- or late-term effects of cancer and its treatment?
Only options:<br>- "Anorexia/Loss of Appetite"<br>- "Assistive Equipment"<br>- "Cardiotoxicity"<br>- "GI Symptoms" (This includes constipation, diarrhea, nausea, and vomiting which were separate symptoms in extraction tool)<br>- "Infertility"<br>- "Lung Toxicity"<br>- "Lymphedema"<br>- "Malnutrition/Cachexia"<br>- "Mobility/Musculoskeletal Issues"<br>- "Neurologic/Cognitive Deficits"<br>- "Neuropathy"<br>- "Obesity"<br>- "Pain"<br>- "Sensory Changes" (tinnitus, ringing in the ears, changes to taste, changes in ability to smell, vision changes)<br>- "Sexual Health"<br>- "Sleeping Difficulty"<br>- "Weakness"<br>- "Other" (No need for further specification here)<br><br>If reviewer indicated something else, please check in original study, and enter one of the above options as appropriate<br><br>If more than one indicated, please separate by only a comma<br><br>Example: "Lung Toxicity,Cardiotoxicity"	Either "Yes" or "No"<br><br>If reviewer indicated "Other" or "Unsure", please check in original study, and enter either "Yes" or "No" in this column

If yes, which *PSYCHOSOCIAL* effects are discussed?	Does the article talk about *CARE COORDINATION* between different stakeholders (including specialist and non-specialist care providers)?	If yes, what is the model of *CARE COORDINATION* discussed in the article?
Only options:<br>- "Anxiety"<br>- "Body Image Alterations"<br>- "Community Rejection"<br>- "Depression"<br>- "Fear of Death"<br>- "Medical Debt/Financial Toxicity"<br>- "Return to Work Difficulty"<br>- "Spousal Abandonment"<br>- "Stigma"<br>- "Other" (No need for further specification here)<br><br>If reviewer indicated something else, please check in original study, and enter one of the above options as appropriate<br><br>If more than one indicated, please separate by only a comma<br><br>Example: "Anxiety,Depression,Medical Debt/Financial Toxicity"	Either "Yes" or "No"<br><br>If reviewer indicated "Other" or "Unsure", please check in original study, and enter either "Yes" or "No" in this column	Only options:<br>- "Between oncology specialist and primary care providers"<br>- "Between oncology specialist and palliative care provider"<br>- "Between oncology specialist and other practitioners"<br>- "Between oncology specialist and community leaders"<br>- "Other" (If doesn't fit in any of previous options)<br><br>Answer should be entered here only if previous column answer is "Yes"<br><br>Otherwise, the value here should be "N/A"

What key groups are mentioned in relation to survivorship care delivery or interventions?	Is there an intervention in this study?	If yes, summarize the intervention in one sentence maximum. Provide page numbers, if applicable.
Only options:<br>- "Cancer Centers"<br>- "Caregivers"<br>- "Community Health Workers (CHW)"<br>- "Complementary & Alternative Medicine Practitioners"<br>- "Faith Based Organizations (FBOs)"<br>- "Home Based Care Providers"<br>- "Non Governmental Organizations (NGOs)"<br>- "Nurses"<br>- "Patient Navigators"<br>- "Peer Survivors"<br>- "Policymakers"<br>- "Primary Care or General Practitioners"<br>- "Other"<br>- "None"<br><br>If more than one indicated, please separate by only a comma	Either "Yes" or "No"<br><br>If reviewer indicated "Other" or "Unsure", please check in original study, and enter either "Yes" or "No" in this column	Definition should be entered here only if previous column answer is "Yes"<br><br>Otherwise, the value here should be "N/A"<br><br>An "Intervention" is an event that happened and the study examined the effect of that intervention<br><br>Conducting a survey is NOT an intervention<br>Discussing the effect the study may have on cancer care is NOT an intervention

Was quality of life (QOL) measured in the study via a questionnaire or survey?	If yes, was measuring quality of life the primary purpose of the study?	Other comments
Either "Yes" or "No"<br><br>If reviewer indicated "Other" or "Unsure", please check in original study, and enter either "Yes" or "No" in this column	Either "Yes" or "No" ONLY if the answer to the previous question in column AB is "Yes"<br><br>Otherwise, the answer must be "N/A"<br><br>If reviewer indicated "Other" or "Unsure", please check in original study, and enter either "Yes", "No", or "N/A" in this column as appropriate	This is one of the fields where many reviewers indicated whether a specific study must be included or excluded
