# Supplementary material for: A scoping review exploring cancer survivorship in Africa: 2011 to 2024
Source: J Cancer Surviv. 2025 Apr 25;20(Suppl 1):1127–37. doi: 10.1007/s11764-025-01805-5 (PMC13676652; doi:10.1007/s11764-025-01805-5)
Supplement: Supplementary file 4 [file 11764_2025_1805_MOESM4_ESM.pdf]

Online Resources 4–6

Title: A scoping review exploring cancer survivorship care in Africa: 2011 to 2024

Journal: *Journal of Cancer Survivorship*

Authors:

Tonia Onyeka, MBBS, MSc, FWACS, Professor, Department of Anaesthesia/Pain & Palliative Care Unit/College of Medicine, University of Nigeria/IVAN Research Institute, Ituku-Ozalla Campus, Enugu, Nigeria

Abdulhafiz Zakieh, MD, Clinical Fellow, Pediatric Oncology Branch, National Cancer Institute, National Institutes of Health, Bethesda, Maryland, USA

Isaiah Gitonga, MPH, Doctoral Researcher, Maynooth University, Maynooth, Co. Kildare, Ireland

Goodluck Nchasi, MD, Medical Doctor, Bugando Medical Center, Mwanza, Tanzania

Md Ahasanur Rahman, PhD, Postdoctoral Fellow, National Cancer Institute, Frederick, Maryland, USA

Nikki Prattipati, MScGH, Independent Researcher, Birmingham, Alabama USA; Current Affiliation: Boston University School of Medicine, Boston, Massachusetts, USA

Alicia A. Livinski, MPH, MA, Biomedical Librarian, National Institutes of Health Library, Office of Research Services, Office of the Director, National Institutes of Health, Bethesda, Maryland, USA

Timiya S. Nolan, PhD, APRN-CNP, FAAN, Associate Professor, University of Alabama at Birmingham, Birmingham, Alabama, USA

Mishka Kohli Cira, MPH, Public Health Advisor, Center for Global Health, National Cancer Institute, Rockville, Maryland, USA

Corresponding Author:
Mishka Kohli Cira, MPH
Public Health Advisor
Center for Global Health, National Cancer Institute
Rockville, Maryland, USA
Email: Mishka.Cira@nih.gov

Online Resource 4: Number of Publications from 2011 to 2024

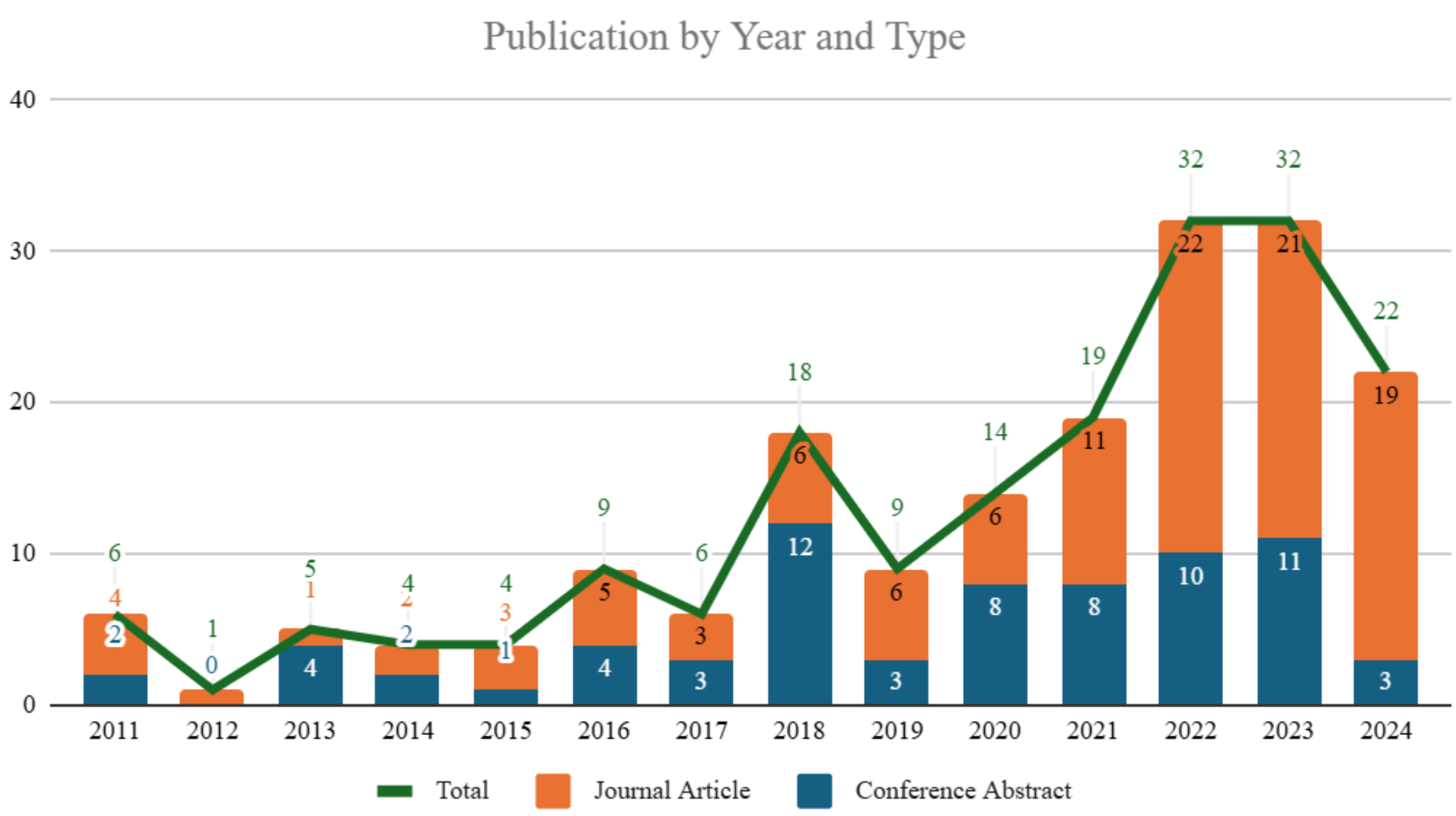

Footnote: Total Studies=181. Searches conducted in August 2024.

Online Resource 5: Distribution of Research Studies Across Different Settings

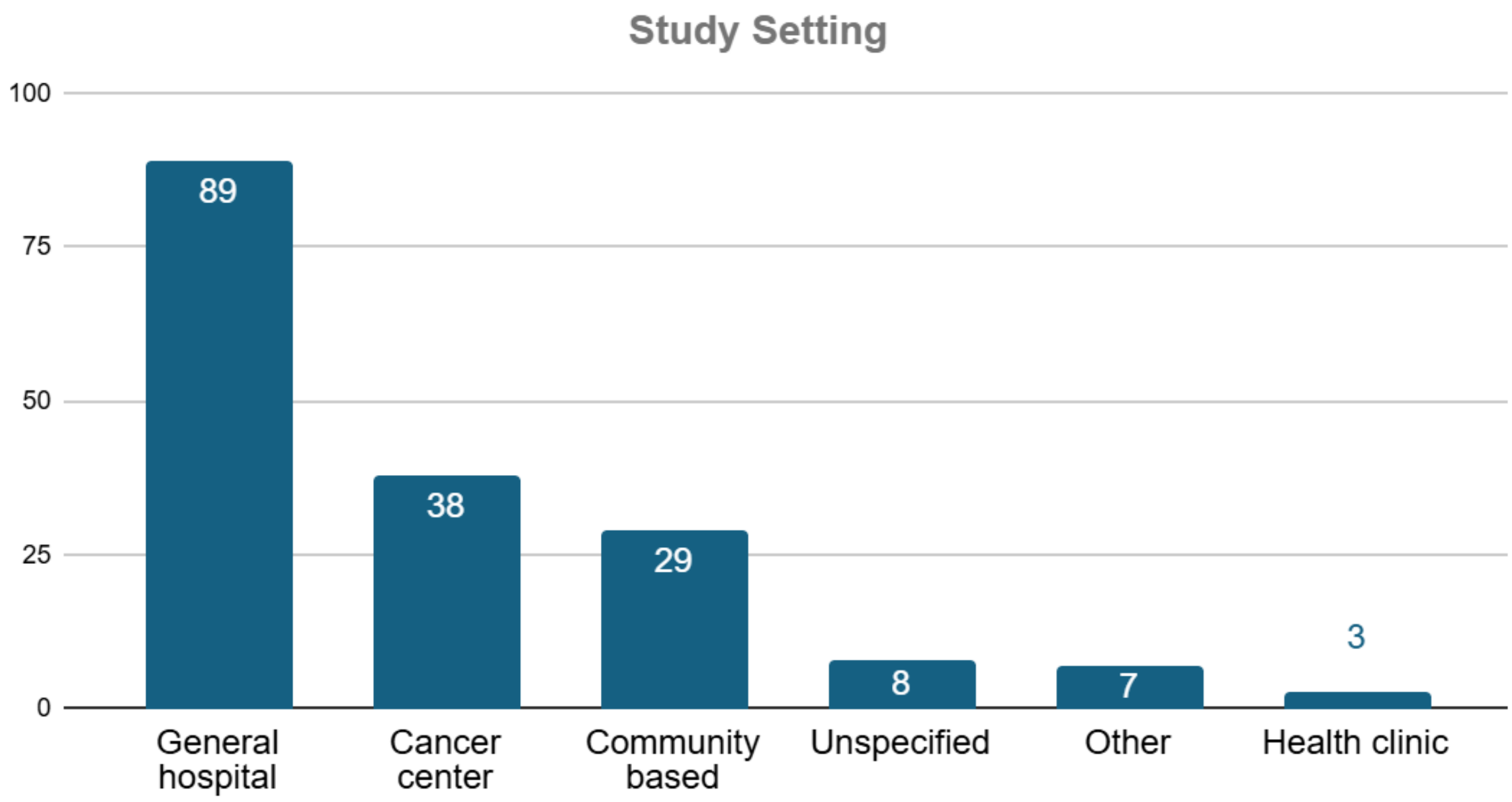

Footnote: Total Studies=181. Other=Research studies that did not fit in any of the listed settings.

Online Resource 6: Number of Studies that Addressed Survivorship by Cancer Type

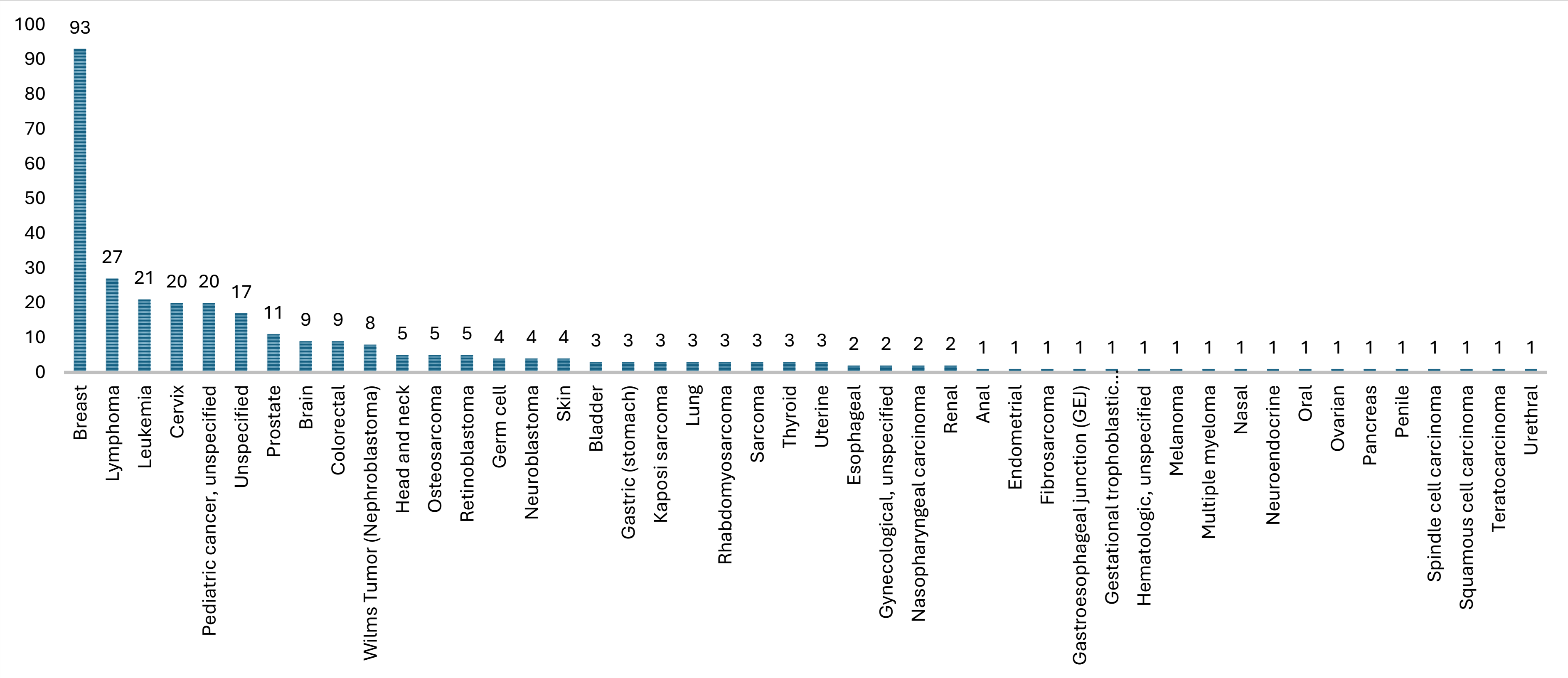

Footnote: Total Studies=181. GU=genitourinary; NOS=not otherwise specified; GI=gastrointestinal; Unspecified=specific type of cancer was not mentioned; Other included: cancer type was specified but was not any of the shown categories.
